# Supplementary material for: Structural basis of mechano-chemical coupling by the mitotic kinesin KIF14
Source: Nat Commun. 2021 Jun 15;12:3637. doi: 10.1038/s41467-021-23581-3 (PMC8206134; doi:10.1038/s41467-021-23581-3)
Supplement: Supplementary file 1 — Supplementary Information [file 41467_2021_23581_MOESM1_ESM.pdf]

| | Semi-Closed | Open | | | | | | | | Open* | | | | | | | | Closed | | | | | | | |
|---|---|---|---|---|---|---|---|---|---|---|---|---|---|---|---|---|---|---|---|---|---|---|---|---|---|
| | K-ADP | MT-K743-ADP | MT-K748-ADP | MT-K755-ADP | MT-K772-ADP | MT-K735-Apo | MT-K743-Apo | MT-K755-Apo | MT-K772-Apo | MT-K735-ANP | MT-K743-ANP-O | MT-K755-ANP-L | MT-K772-ANP-L | MT-K735-AAF | MT-K743-AAF-O | MT-K755-AAF-L | MT-K772-AAF-L | MT-K743-ANP-C | MT-K748-ANP | MT-K755-ANP-T | MT-K772-ANP-T | MT-K743-AAF-C | MT-K748-AAF | MT-K755-AAF-T | MT-K772-AAF-T |
| K-ADP | 0.00 | 1.31 | 1.36 | 1.40 | 1.39 | 1.35 | 1.35 | 1.60 | 1.59 | 1.19 | 1.26 | 1.41 | 1.41 | 1.27 | 1.33 | 1.41 | 1.47 | 2.07 | 2.05 | 2.08 | 1.90 | 2.11 | 2.13 | 2.11 | 2.15 |
| MT-K743-ADP | 1.31 | 0.00 | 0.62 | 0.74 | 0.79 | 0.67 | 0.76 | 0.91 | 0.90 | 1.02 | 0.72 | 0.92 | 1.12 | 0.71 | 0.66 | 0.91 | 0.98 | 2.46 | 2.45 | 2.53 | 2.34 | 2.54 | 2.54 | 2.56 | 2.60 |
| MT-K748-ADP | 1.36 | 0.62 | 0.00 | 0.82 | 0.77 | 0.53 | 0.64 | 0.97 | 0.96 | 1.09 | 0.68 | 0.92 | 1.11 | 0.73 | 0.68 | 0.92 | 0.99 | 2.47 | 2.46 | 2.55 | 2.35 | 2.55 | 2.55 | 2.56 | 2.61 |
| MT-K755-ADP | 1.40 | 0.74 | 0.82 | 0.00 | 0.66 | 0.86 | 0.91 | 0.67 | 0.91 | 1.13 | 0.89 | 0.85 | 1.07 | 0.91 | 0.85 | 0.81 | 0.90 | 2.53 | 2.53 | 2.60 | 2.41 | 2.61 | 2.61 | 2.62 | 2.66 |
| MT-K772-ADP | 1.39 | 0.79 | 0.77 | 0.66 | 0.00 | 0.79 | 0.82 | 0.73 | 0.89 | 1.07 | 0.84 | 0.85 | 1.10 | 0.90 | 0.82 | 0.84 | 0.91 | 2.54 | 2.53 | 2.60 | 2.41 | 2.62 | 2.61 | 2.63 | 2.68 |
| MT-K735-Apo | 1.35 | 0.67 | 0.53 | 0.86 | 0.79 | 0.00 | 0.60 | 0.98 | 0.94 | 1.02 | 0.63 | 0.93 | 1.14 | 0.69 | 0.73 | 0.95 | 1.01 | 2.46 | 2.44 | 2.54 | 2.33 | 2.53 | 2.53 | 2.55 | 2.60 |
| MT-K743-Apo | 1.35 | 0.76 | 0.64 | 0.91 | 0.82 | 0.60 | 0.00 | 1.02 | 0.99 | 1.02 | 0.68 | 0.93 | 1.14 | 0.80 | 0.79 | 0.99 | 1.05 | 2.55 | 2.54 | 2.61 | 2.42 | 2.63 | 2.62 | 2.63 | 2.68 |
| MT-K755-Apo | 1.60 | 0.91 | 0.97 | 0.67 | 0.73 | 0.98 | 1.02 | 0.00 | 0.86 | 1.27 | 1.04 | 0.94 | 1.16 | 1.08 | 1.00 | 0.91 | 1.00 | 2.63 | 2.61 | 2.68 | 2.52 | 2.71 | 2.70 | 2.72 | 2.77 |
| MT-K772-Apo | 1.59 | 0.90 | 0.96 | 0.91 | 0.89 | 0.94 | 0.99 | 0.86 | 0.00 | 1.22 | 1.00 | 1.08 | 1.31 | 1.01 | 1.02 | 1.09 | 1.18 | 2.61 | 2.61 | 2.68 | 2.48 | 2.71 | 2.69 | 2.71 | 2.74 |
| MT-K735-ANP | 1.19 | 1.02 | 1.09 | 1.13 | 1.07 | 1.02 | 1.02 | 1.27 | 1.22 | 0.00 | 0.98 | 1.20 | 1.28 | 1.05 | 1.10 | 1.22 | 1.27 | 2.12 | 2.11 | 2.16 | 2.00 | 2.20 | 2.18 | 2.22 | 2.25 |
| MT-K743-ANP-O | 1.26 | 0.72 | 0.68 | 0.89 | 0.84 | 0.63 | 0.68 | 1.04 | 1.00 | 0.98 | 0.00 | 0.86 | 1.04 | 0.68 | 0.68 | 0.90 | 1.00 | 2.39 | 2.36 | 2.43 | 2.23 | 2.45 | 2.45 | 2.46 | 2.49 |
| MT-K755-ANP-L | 1.41 | 0.92 | 0.92 | 0.85 | 0.85 | 0.93 | 0.93 | 0.94 | 1.08 | 1.20 | 0.86 | 0.00 | 0.70 | 0.91 | 0.91 | 0.50 | 0.67 | 2.49 | 2.49 | 2.54 | 2.32 | 2.55 | 2.54 | 2.56 | 2.60 |
| MT-K772-ANP-L | 1.41 | 1.12 | 1.11 | 1.07 | 1.10 | 1.14 | 1.14 | 1.16 | 1.31 | 1.28 | 1.04 | 0.70 | 0.00 | 1.12 | 1.12 | 0.75 | 0.89 | 2.33 | 2.33 | 2.38 | 2.16 | 2.39 | 2.39 | 2.40 | 2.44 |
| MT-K735-AAF | 1.27 | 0.71 | 0.73 | 0.91 | 0.90 | 0.69 | 0.80 | 1.08 | 1.01 | 1.05 | 0.68 | 0.91 | 1.12 | 0.00 | 0.64 | 0.89 | 0.96 | 2.39 | 2.36 | 2.44 | 2.24 | 2.45 | 2.45 | 2.46 | 2.51 |
| MT-K743-AAF-O | 1.33 | 0.66 | 0.68 | 0.85 | 0.82 | 0.73 | 0.79 | 1.00 | 1.02 | 1.10 | 0.68 | 0.91 | 1.12 | 0.64 | 0.00 | 0.82 | 0.88 | 2.47 | 2.44 | 2.52 | 2.33 | 2.53 | 2.55 | 2.54 | 2.59 |
| MT-K755-AAF-L | 1.41 | 0.91 | 0.92 | 0.81 | 0.84 | 0.95 | 0.99 | 0.91 | 1.09 | 1.22 | 0.90 | 0.50 | 0.75 | 0.89 | 0.82 | 0.00 | 0.52 | 2.50 | 2.48 | 2.54 | 2.34 | 2.56 | 2.56 | 2.56 | 2.60 |
| MT-K772-AAF-L | 1.47 | 0.98 | 0.99 | 0.90 | 0.91 | 1.01 | 1.05 | 1.00 | 1.18 | 1.27 | 1.00 | 0.67 | 0.89 | 0.96 | 0.88 | 0.52 | 0.00 | 2.52 | 2.50 | 2.55 | 2.37 | 2.57 | 2.58 | 2.59 | 2.63 |
| MT-K743-ANP-C | 2.07 | 2.46 | 2.47 | 2.53 | 2.54 | 2.46 | 2.55 | 2.63 | 2.61 | 2.12 | 2.39 | 2.49 | 2.33 | 2.39 | 2.47 | 2.50 | 2.52 | 0.00 | 0.69 | 0.70 | 0.97 | 0.63 | 0.71 | 0.68 | 0.70 |
| MT-K748-ANP | 2.05 | 2.45 | 2.46 | 2.53 | 2.53 | 2.44 | 2.54 | 2.61 | 2.61 | 2.11 | 2.36 | 2.49 | 2.33 | 2.36 | 2.44 | 2.48 | 2.50 | 0.69 | 0.00 | 0.58 | 0.95 | 0.65 | 0.58 | 0.62 | 0.63 |
| MT-K755-ANP-T | 2.08 | 2.53 | 2.55 | 2.60 | 2.60 | 2.54 | 2.61 | 2.68 | 2.68 | 2.16 | 2.43 | 2.54 | 2.38 | 2.44 | 2.52 | 2.54 | 2.55 | 0.70 | 0.58 | 0.00 | 0.85 | 0.66 | 0.60 | 0.53 | 0.52 |
| MT-K772-ANP-T | 1.90 | 2.34 | 2.35 | 2.41 | 2.41 | 2.33 | 2.42 | 2.52 | 2.48 | 2.00 | 2.23 | 2.32 | 2.16 | 2.24 | 2.33 | 2.34 | 2.37 | 0.97 | 0.95 | 0.85 | 0.00 | 0.97 | 0.95 | 0.89 | 0.89 |
| MT-K743-AAF-C | 2.11 | 2.54 | 2.55 | 2.61 | 2.62 | 2.53 | 2.63 | 2.71 | 2.71 | 2.20 | 2.45 | 2.55 | 2.39 | 2.45 | 2.53 | 2.56 | 2.57 | 0.63 | 0.65 | 0.66 | 0.97 | 0.00 | 0.60 | 0.53 | 0.55 |
| MT-K748-AAF | 2.13 | 2.54 | 2.55 | 2.61 | 2.61 | 2.53 | 2.62 | 2.70 | 2.69 | 2.18 | 2.45 | 2.54 | 2.39 | 2.45 | 2.55 | 2.56 | 2.58 | 0.71 | 0.58 | 0.60 | 0.95 | 0.60 | 0.00 | 0.50 | 0.55 |
| MT-K755-AAF-T | 2.11 | 2.56 | 2.56 | 2.62 | 2.63 | 2.55 | 2.63 | 2.72 | 2.71 | 2.22 | 2.46 | 2.56 | 2.40 | 2.46 | 2.54 | 2.56 | 2.59 | 0.68 | 0.62 | 0.53 | 0.89 | 0.53 | 0.50 | 0.00 | 0.42 |
| MT-K772-AAF-T | 2.15 | 2.60 | 2.61 | 2.66 | 2.68 | 2.60 | 2.68 | 2.77 | 2.74 | 2.25 | 2.49 | 2.60 | 2.44 | 2.51 | 2.59 | 2.60 | 2.63 | 0.70 | 0.63 | 0.52 | 0.89 | 0.55 | 0.55 | 0.42 | 0.00 |

**Supplementary Table 1. KIF14 motor domain structures pairwise comparison.** The table shows the root mean square distance (RMSD) in Å, after optimal alignment of equivalent Cα carbons of the KIF14 motor domain of the two complexes specified in the corresponding row and column. Only residues resolved in all the structures were used in the alignment and RMSD calculations. This excludes part of the tubulin interacting loops and the tips of the switch loops not visible in the microtubule-unbound crystal structure, as well as the neck-linker which is only visible in some microtubule-bound structures. Table cells are color coded according to their RMSD values.

| Dataset | Microscope used | Pixel Size (Å) | Box size helical map (pixels) | Magnification anisotropy % [a] | Magnification anisotropy angle [a] | Per particle CTF refinement | 15R particle cleaning | Box size (pixels) |
|---|---|---|---|---|---|---|---|---|
| 735-ANP | Krios 1 | 1.073 | 512 | n.c. | n.c. | N | N | 320 |
| 735-AAF | Krios 1 | 0.831 | 664 | 0.33 | 88.7 | Y | N | 416 |
| 735-Apo | Krios 1 | 1.073 | 512 | n.c. | n.c. | N | N | 320 |
| 743-ANP | Krios 2 | 0.849 | 664 | 1.45 | 1 | Y | Y | 416 |
| 743-AAF | Krios 3 | 0.825 | 664 | 0.54 | 48.4 | Y | Y | 416 |
| 743-ADP | Krios 1 | 0.828 | 664 | 1.06 | 87.7 | Y | Y | 416 |
| 743-Apo | Krios 2 | 0.848 | 664 | 1.69 | 179 | Y | Y | 416 |
| 748-ANP | Krios 1 | 0.828 | 664 | 1.07 | 89.8 | Y | Y | 416 |
| 748-AAF | Krios 3 | 0.825 | 664 | 0.52 | 47.4 | Y | Y | 416 |
| 748-ADP | Krios 2 | 0.849 | 664 | 1.46 | 175.9 | Y | Y | 416 |
| 755-ANP | Krios 1 | 1.072 | 512 | n.c. | n.c. | Y | Y | 320 |
| 755-AAF | Krios 2 | 0.848 | 664 | 1.6 | 179.2 | Y* | Y* | 416 |
| 755-ADP | Krios 1 | 0.831 | 664 | 0.35 | 70 | N | N | 416 |
| 755-Apo | Krios 2 | 1.088 | 512 | 1.5 | 171.7 | N | N | 320 |
| 772-ANP | Krios 1 | 1.073 | 512 | n.c. | n.c. | N | N | 320 |
| 772-AAF | Krios 3 | 0.826 | 664 | 0.44 | 49.6 | Y | Y | 414 |
| 772-ADP | Krios 2 | 0.849 | 664 | 1.46 | 3.6 | N | N | 416 |
| 772-Apo | Krios 2 | 0.850 | 664 | 1.25 | 3.1 | N | N | 416 |

**Supplementary Table 2. Extra data collection and refinement parameters.**

Notes

[a] Given when estimated and corrected for a given dataset.

n.c.: magnification anisotropy not corrected.

* Dataset for which this step was performed after the final helical reconstruction and before the helical assembly subunit refinement and classification (HASRC) procedure.

Motor Domain

Neck Linker

Coiled Coil 1 (CC1)

```
                                                                     391
mmKIF14   344 ASPRVKPRPKSSLFANKRESSRESTLPPEENSLVQKTFTEPDSLKVENSQVTVAVRVRPF
hsKIF14   309 SNLQVKQRPKSSFLANKQERSAENTILPEEETVVQNTSAGKDPLKVENSQVTVAVRVRPF

mmKIF14   404 SKREKTEKASQVVFTNGEEITVEHPDMKQVYSFIYDVSFWSFDECHPGYASQTTVYETLA
hsKIF14   369 TKREKIEKASQVVFMSGKEITVEHPDTKQVYNFIYDVSFWSFDECHPHYASQTTVYEKLA

mmKIF14   464 APLLDRAFEGYNTCLFAYGQTGSGKSYTMMGLNEEPGIIPRFCEDLFAQIAKKQTSEVSY
hsKIF14   429 APLLERAFEGFNTCLFAYGQTGSGKSYTMMGFSEEPGIIPRFCEDLFSQVARKQTQEVSY

mmKIF14   524 HLEMSFFEVYNEKIHDLLVCKGENGQRKQPLRAREHPVSGPYVEGLSMNVVSSYSDIQSW
hsKIF14   489 HIEMSFFEVYNEKIHDLLVCKDENGQRKQPLRVREHPVYGPYVEALSMNIVSSYADIQSW

mmKIF14   584 LELGNKQRATAATGMNDKSSRSHSVFTLVMTQTKTEVVEGEEHDHRITSRINLVDLAGSE
hsKIF14   549 LELGNKQRATAATGMNDKSSRSHSVFTLVMTQTKTEFVEGEEHDHRITSRINLIDLAGSE

mmKIF14   644 RCSTAHSSGQRLKEGVSINKSLLTLGKVISALSEQANGKRVFIPYRESTLTWLLKESLGG
hsKIF14   609 RCSTAHTNGDRLKEGVSINKSLLTLGKVISALSEQANQRSVFIPYRESVLTWLLKESLGG

                                         735         743    748       755
mmKIF14   704 NSKTAMIATVSPAASNIEETLSTLRYATQARLIVNIAKVNEDMNAKLIRELKAEIEKLKA
hsKIF14   669 NSKTAMIATISPAASNIEETLSTLRYANQARLIVNIAKVNEDMNAKLIRELKAEIAKLKA

                       772
mmKIF14   764 AQRSNRNIDPERYRLCRQEITSLRMKLHQQERDMAEIQRVWKEKFEQAEKRKLQETKELQ
hsKIF14   729 AQRNSRNIDPERYRLCRQEITSLRMKLHQQERDMAEMQRVWKEKFEQAEKRKLQETKELQ
```

**Supplementary Fig. 1. Mouse and human KIF14 amino-acid sequence alignment.** The figure shows the sequence of the motor domain and flaking regions of mouse and human KIF14 (Uniprot accession codes L0N7N1 and Q15058 respectively). Numbers above the sequences indicate the first and last residues of the five KIF14 constructs used.

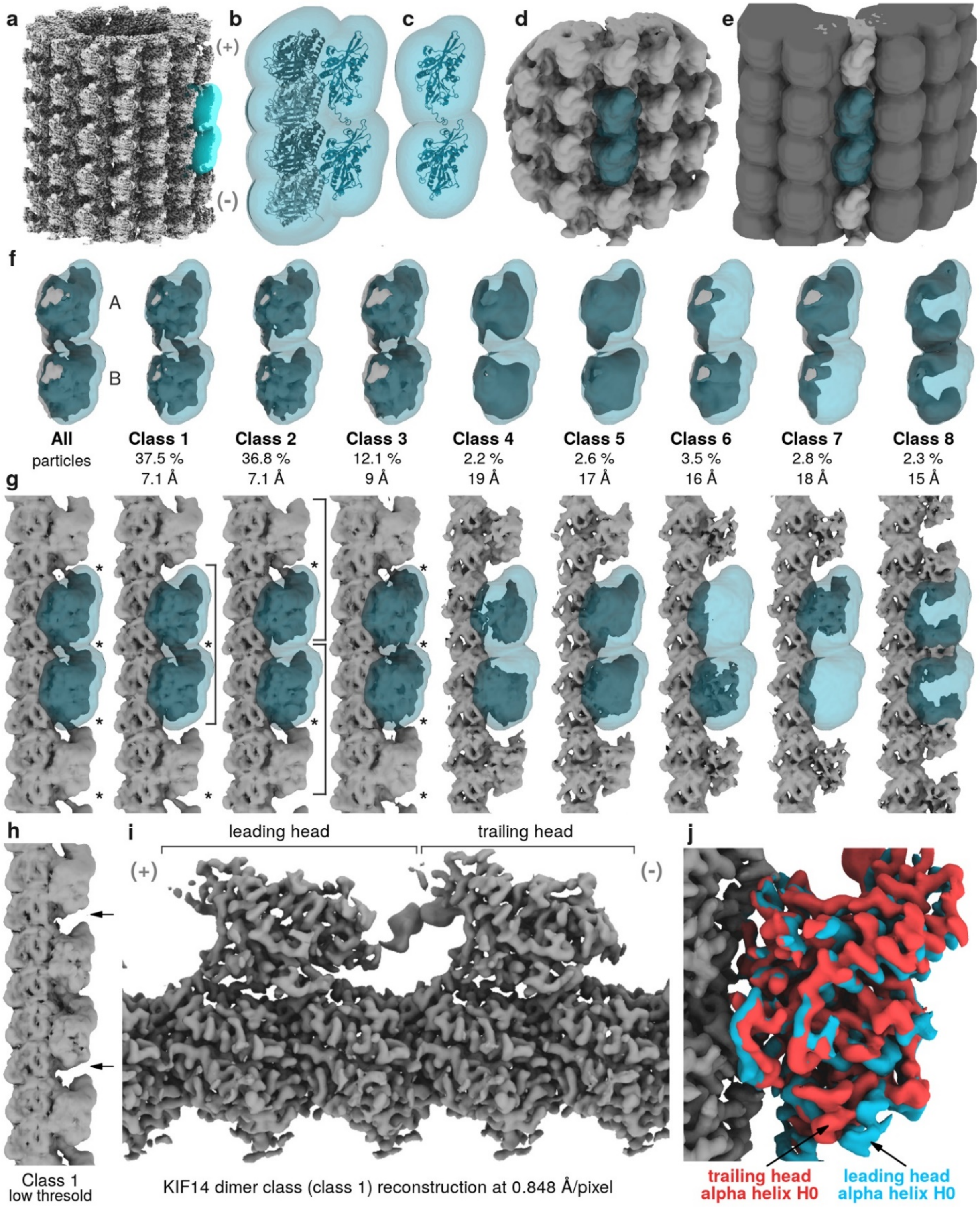


**Supplementary Fig. 2. Helical 3D reconstruction with subunit refinement and classification (HASRC): Kinesin two-heads-bound example (a)** Helical reconstruction of MT-K755-AAF dataset with $mask_{full}$ displayed as a semitransparent blue surface. **(b)** $mask_{full}$ (threshold 0.5) with underlying model displayed as ribbons. This mask was used for partial signal subtraction to improve the alignment locally. **(c)** $mask_{kin}$ (threshold 0.5) with underlying model displayed as ribbons. This mask was used for partial signal subtraction to isolate the kinesin signal before 3D classification. **(d)** Reconstruction with all the particles without partial signal subtraction at 3.5 Å/pixel after the asymmetric refinement step with partial signal subtraction. $mask_{classif}$ displayed as a semitransparent blue surface. **(e)** Similar view as (d) but with the soft mask used to isolate the

kinesin signal of the central protofilament for panel (g). **(f)** The eight 3D class averages as well as a reconstruction with all the particles used in the 3D classification ("All particles" on left most panel). The mask used for classification $mask_{classif}$ is overlayed and displayed as a semitransparent blue surface. For each class, the percentage of particles assigned to this class and the resolution of the class average is given. Pixel size is 3.5 Å/pixel. The interpretation of these classes is facilitated by the reconstructions in (g). The 2 kinesin binding sites are labeled A and B on the (+) end and (-) end respectively. **(g)** Masked reconstructions of all the particles and classes shown in (f) using particles without partial signal subtraction. To facilitate interpretation, these reconstructions (like the one in (d)) were masked with the mask in (e) to isolate the kinesin signal of the central protofilament. Pixel size is the same as in (f). The mask used for classification $mask_{classif}$ is overlayed and displayed as a semitransparent blue surface. Before classification ("All particles"), all kinesin binding sites show densities connecting them (labeled with *) indicative of connected heads of kinesin dimers. Class 1: Note a clear connection between kinesins on site A and B and not with other kinesins along the protofilament (even at high threshold as shown in (h)). This shows that this class corresponds to an isolated dimer. The dimer is highlighted by a square bracket. Class 2: This class is centered between 2 kinesin dimers as evidenced by the connectivity pattern (marked with *). The two dimers are highlighted by square brackets. Such a class is expected from the methodology which samples the microtubule lattice between every kinesin binding site. In that class, site A is populated by a trailing head from a dimer on the (+) end while site B contains a leading head from a dimer on the (-) end of the reconstructed protofilament. Class 3: Residual connectivity between all the kinesin sites indicates that this class contains particles for which the kinesin dimeric state is not well separated. Class 4 & 5: Low resolution class averages for which the kinesin state cannot be assessed. Classes 6 & 7: Cases where the site A or B respectively is not occupied by a kinesin motor. Class 8: Protofilament out of register (tubulin alpha and beta mismatched). Classes 1 to 8: The class averages and reconstructions show that the kinesin signal is essentially explained by a series of kinesin in dimeric state on the microtubule with a few low-resolution kinesins and empty kinesin binding sites. **(h)** Lack of visible connecting densities in Class 1 between kinesins outside the central dimer (arrows) even contouring at low iso-density threshold. **(i)** Reconstruction of the dimer class (class 1) at 0.848 Å/pixel. The connection between the head was low pass filtered to 8 Å as explained in Fig.1 and methods. **(j)** Overlay of the densities of the leading head (in blue) and trailing heads (in red) from (i) after alignment on the tubulins. The two heads have two distinct conformations, the leading is in an open state and the trailing head in a closed state. See in particular the two distinct locations corresponding to kinesins alpha helix KH0.

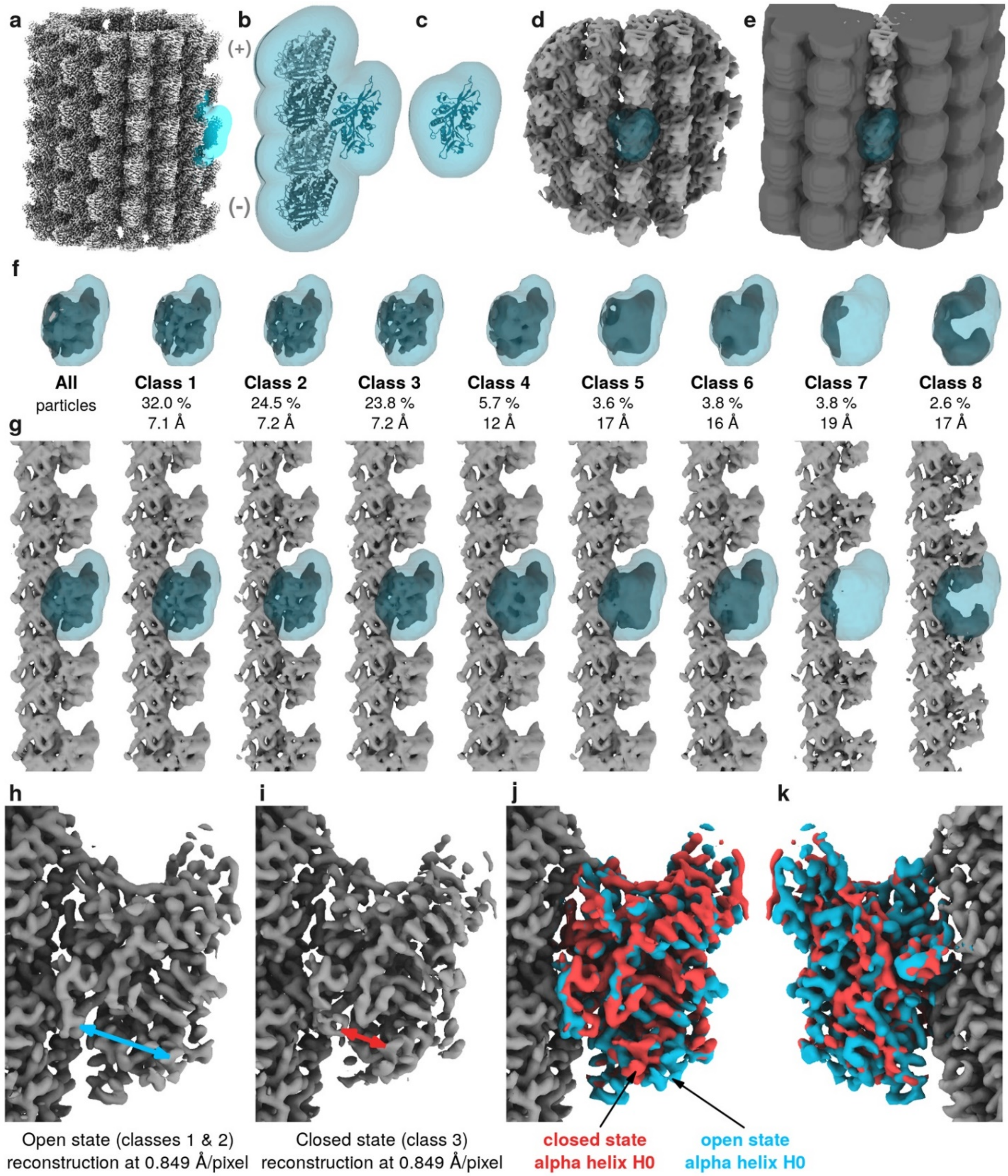


**Supplementary Fig. 3. Helical 3D reconstruction with subunit refinement and classification (HASRC): Kinesin one-head-bound example. (a)** Helical reconstruction of 743-ANP dataset with $mask_{full}$ displayed as a semitransparent blue surface. (**b)** $mask_{full}$ (threshold 0.5) with underlying model displayed as ribbons. This mask was used for partial signal subtraction to improve the alignment locally. **(c)** $mask_{kin}$ (threshold 0.5) with underlying model displayed as ribbons. This mask was used for partial signal subtraction to isolate the kinesin signal before 3D classification. **(d)** Reconstruction with all the particles without partial signal subtraction at 3.5 Å/pixel after the asymmetric refinement step with partial signal subtraction. $mask_{classif}$ displayed as a semitransparent blue surface. **(e)** Similar view as (d) but with the soft mask used to isolate the kinesin signal of

the central protofilament for panel (g). **(f)** The eight 3D class averages as well as a reconstruction with all the particles used in the 3D classification ("All particles" on left most panel). The mask used for classification $mask_{classif}$ is overlayed and displayed as a semitransparent blue surface. For each class, the percentage of particles assigned to this class and the resolution of the class average is given. Pixel size is 3.5 Å/pixel. The interpretation of these classes is facilitated by the reconstructions in (g). **(g)** Masked reconstructions of all the particles and classes shown in (f) using particles without partial signal subtraction. To facilitate the class interpretation, these reconstructions (like the one in (d)) were masked with the mask in (e) to isolate the kinesin signal of the central protofilament. Pixel size is the same as in (f). The mask used for classification $mask_{classif}$ is overlayed and displayed as a semitransparent blue surface. Class 1 & 2: kinesin in an open state (see full size reconstructions in h-k). Class 3: kinesin in a closed state (see h-k). Classes 4-6: These class averages don't show well separated kinesin motor state (mixed open and closed states and/or too low resolution). Class 7: kinesin site not occupied. Class 8: Protofilament out of register (tubulin alpha and beta mismatched). **(h)** Reconstruction of the open state (merge of class 1 and 2) at 0.849 Å/pixel. The distance between switch-2 loop and alpha helix H0 is labeled with a blue double arrow. **(i)** Reconstruction of the closed state (Class 3) at 0.849 Å/pixel. The distance between switch-2 loop and alpha helix H0 is labeled with a red double arrow. **(j)** Overlay of the open and closed state reconstructions from (h) and (i) in blue and red respectively. See in particular the two distinct positions of α-helix-0 (KH0) in the two motor domain structures. **(k)** Same as (j) rotated 180 degrees.

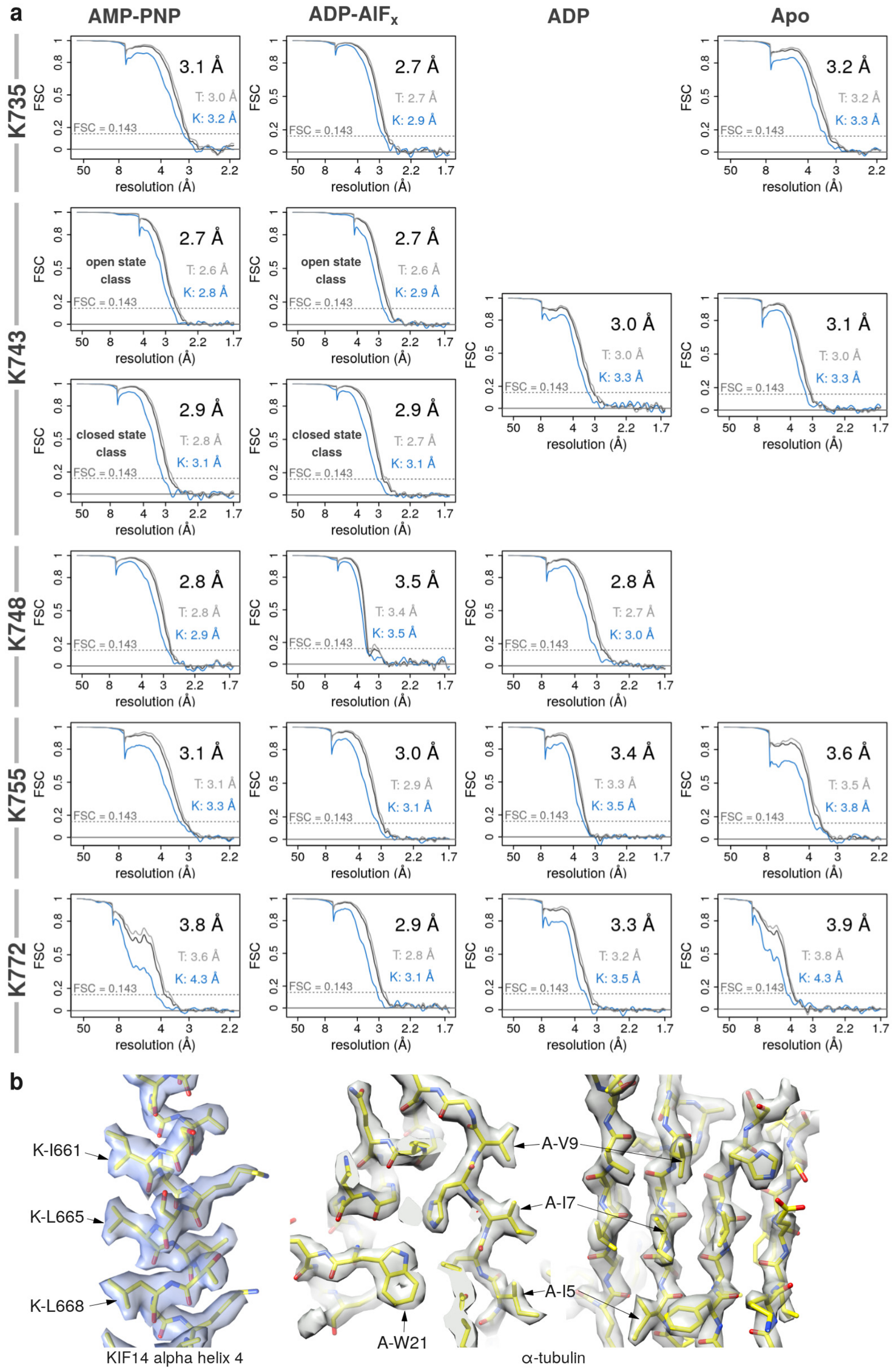


**Supplementary Fig. 4. Resolution estimation. (a)** FSC curves for each of the structures solved. Overall FSC in black, tubulin part FSC in grey and kinesin part FSC in blue. Resolution values ($FSC_{0.143}$) for the overall, tubulin (T) and kinesin (K) parts are indicated. Half maps and masks used to generate the FSC curves are deposited in the EMDB (accession numbers in Tables 1 and 2). **(b)** Iso-density surface representation of the MT-K743-ANP-O class map filtered at the $FSC_{0.143}$ estimated tubulin resolution (2.6 Å) and sharpened with a B-factor of -100 Å$^2$ (deposited as an additional map within the entry EMDB-21946). The leftmost panel shows the KIF14 helix-4 region of the map and the other panels α-tubulin regions. Some KIF14 and α-tubulin residues are indicated (K or A prefix respectively). Note that consistent with the estimated resolution side chains are well resolved as well as many backbone carbonyl groups.

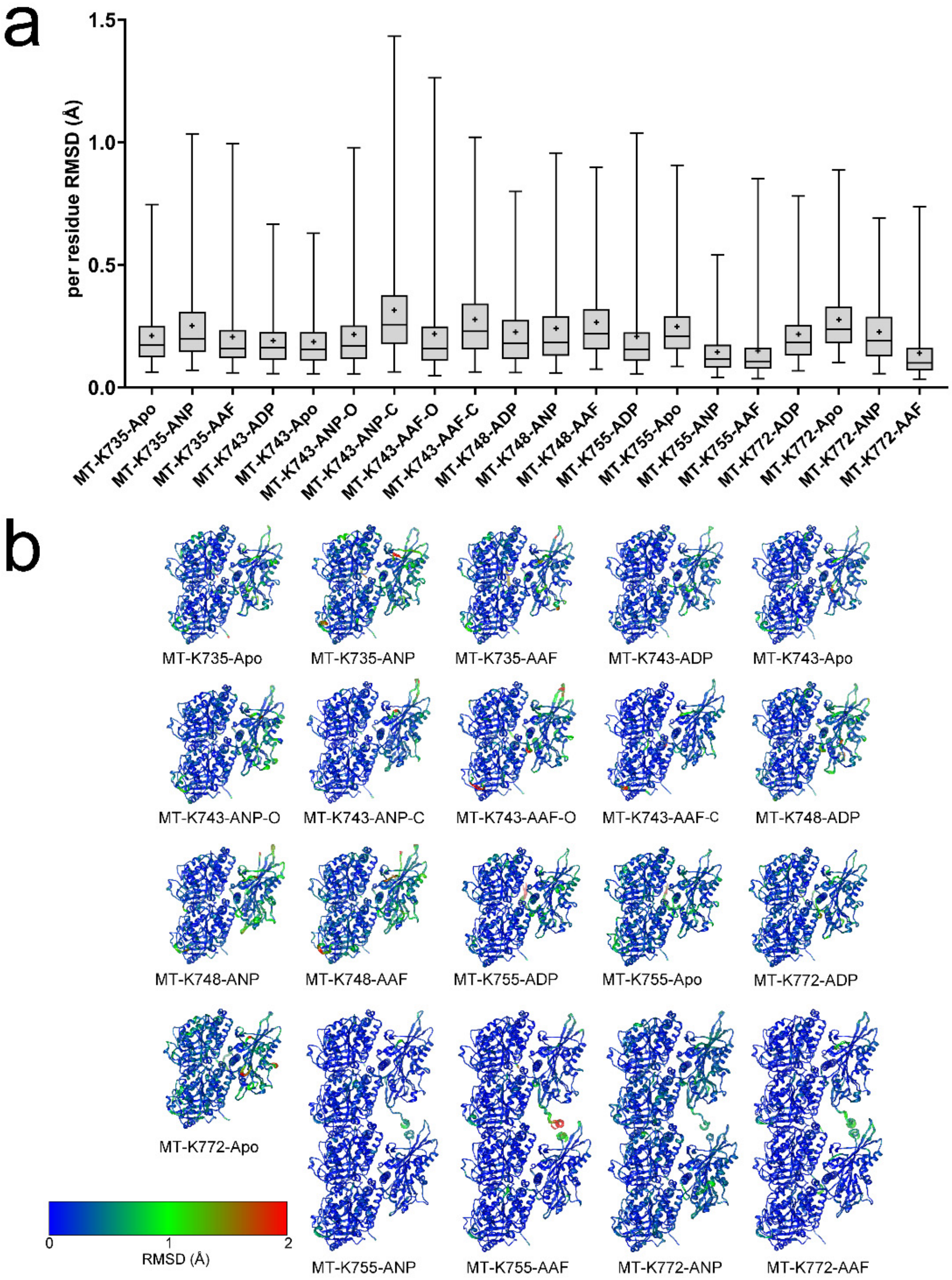


**Supplementary Fig. 5. Atomic models coordinate precision. (a)** Box plot of the distributions of the root mean square deviation (RMSD) between Cα atoms. The number of values in each distribution (n) corresponds to the number of residues in each model structure (one RMSD value per residue). RMSDs per residue were calculated from 20 Rosetta fitted atomic models (methods). Boxes extend from 25 to 75 percentiles of the distributions, whiskers from 1 to 99 percentiles, median position at the inner line and mean at the * symbol. **(b)** Superimposed Rosetta fitted models (20) to each cryo-EM determined density map. Ribbons are color-coded by the Cα RMSD value per residue according to the inset color scale.

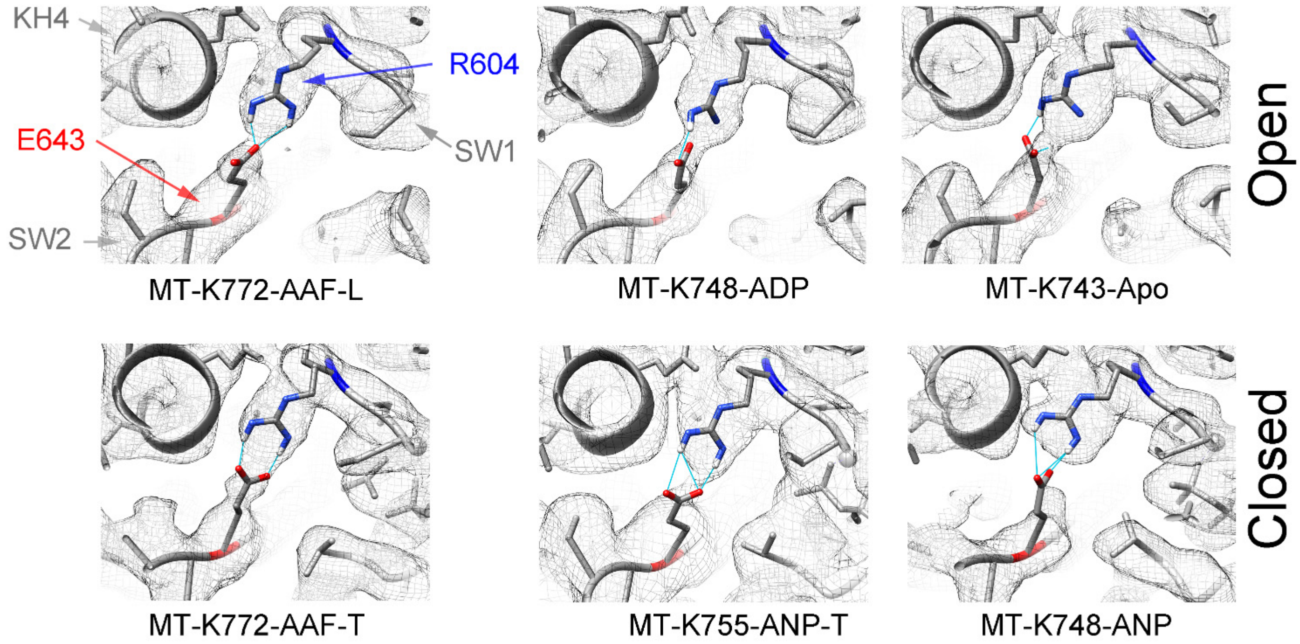


**Supplementary Fig. 6. SW1-SW2 salt bridge.** Densities of residue side chains R604 in SW1 and E643 in SW2, indicative of a salt bridge between these residues were observed in all cryo-EM maps at 3.5 Å resolution or better in the kinesin part of the map, whether they correspond to the open/open* or closed conformations (six examples shown). R604 and E643 atoms in color and the rest of the structure in gray. Blue lines indicate hydrogen bonds identified by UCSF-Chimera findHBond routine. Cryo-EM densities represented as iso-density surface grey mesh.

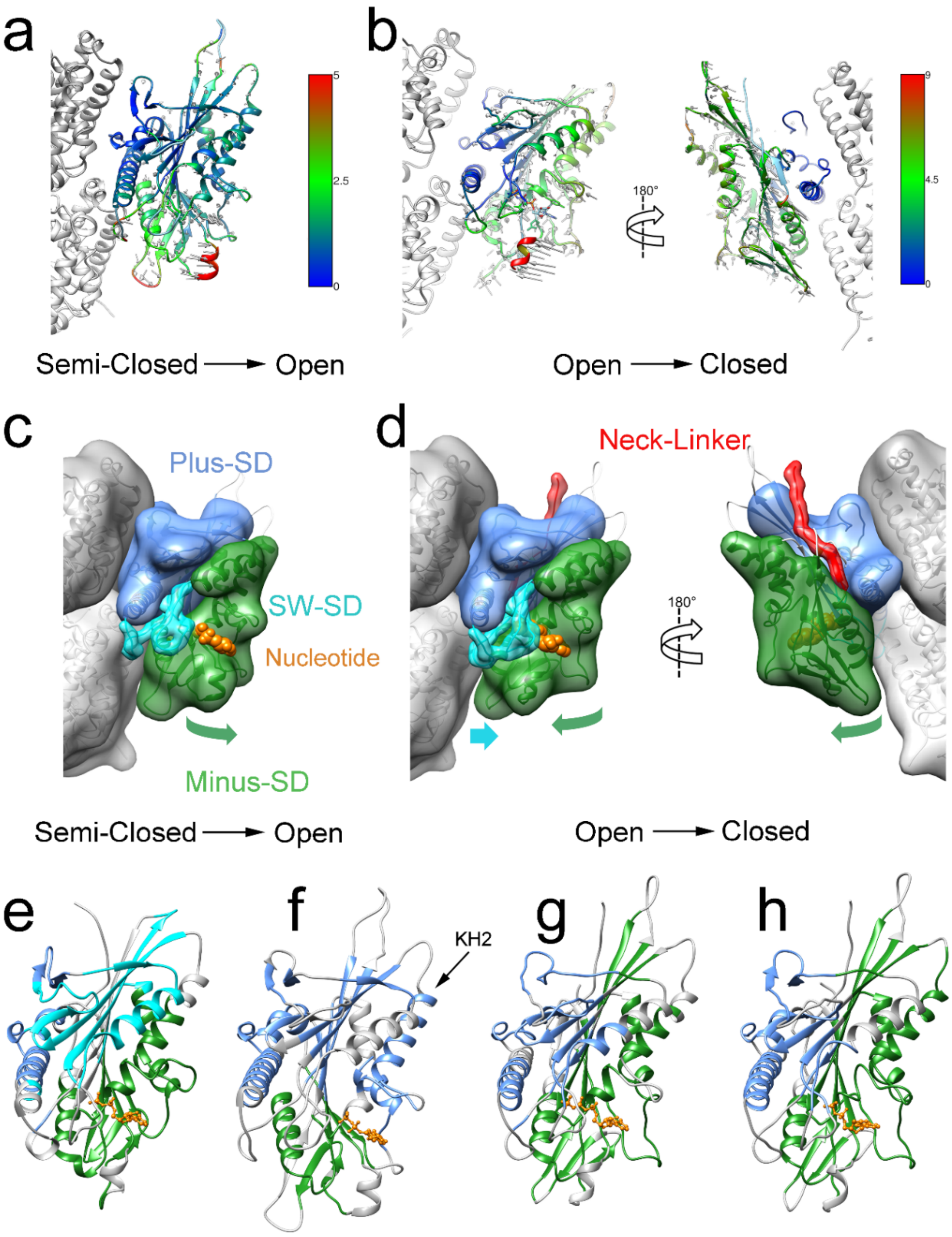


**Supplementary Fig. 7. KIF14 motor subdomains. (a)** KIF14 microtubule bound ADP structure (MT-K748-ADP, open conformation). The arrows indicate the direction of movement of corresponding Cα carbons from the semi-closed (PDB: 4OZQ) to the open (MT-K748-ADP) motor domain conformation. **(b)** Two views of the microtubule bound KIF14 closed conformation. The arrows indicate the direction of movement of corresponding Cα carbons from the open (MT-K748-ADP) to the closed (MT-K748-ANP) motor domain conformations. In (a) and (b) the structures are color coded by the magnitude of the displacement vectors according to the color scale insets. To highlight movements within the motor domain relative to the regions involved in microtubule binding, the structures compared were aligned to the KIF14 areas that interact with β-tubulin in (a) or to the corresponding β-tubulin subunits in (b). Based in the magnitude of the displacements the motor domain structure can be subdivided roughly into two subdomains, a subdomain that remains approximately unchanged (blue colors) and a subdomain that moves relative to the microtubule (green to red).

We named these subdomains plus and minus respectively according to their position in the microtubule complex (all structures are oriented with the microtubule plus end up). The switch loops can be considered a third subdomain as they become ordered with microtubule binding and move slightly relative to the two other subdomains between the open and closed conformations. **(c-d)** Same structures shown in (a-b) with the parts comprising each subdomain (SD) enclosed by semi-transparent surfaces. Plus subdomain in blue, minus in green and the switch subdomain in cyan. The transition between the three major KIF14 motor domain conformations observed, semi-closed, open and closed can be summarized as relative movements between the three defined subdomains. (**c**) Microtubule binding induces ordering of the switch subdomain and a rotation of the minus subdomain relative to the microtubule (clockwise when viewed from the microtubule minus end). This rotation results in an opening of the nucleotide binding pocket (nucleotide in orange). (**d**) Nucleotide analogues that mimic the presence of a γ-phosphate group (AMP-PNP or ADP-$AlF_x$) induce a rotation of the minus subdomain (counterclockwise when viewed from the microtubule minus end) and a slight movement of the switch subdomain towards the nucleotide. These movements result in closure of the nucleotide binding pocket in one side of the motor domain and the opening of a hydrophobic pocket on the other side where the neck-linkers docks. **(e)** Alternative subdomain division proposed by Cao et al.[1] based on the comparison of the structures of tubulin bound kinesin-1 (hKIF5B) in the Apo and ADP-$AlF_x$ states using the program RAPIDO[2]. Three subdomains were identified and named: the microtubule binding subdomain (blue), the P-loop subdomain (green) and the switch I/II subdomain (cyan). (**f-h**) Motor subdomain identified using RAPIDO with the same parameters used by Cao et. al., (low limit = 1) comparing different MT-KIF14 structure pairs: (**f**) K-ADP-4OZQ vs. MT-K748-ADP. **(g)** MT-K748-ADP vs. MT-K748-ANP. **(h)** MT-K743-Apo vs. MT-K748-AAF. In all cases (f-h) the RAPIDO algorithm identifies two subdomains (colored blue and green) similar to the plus and minus subdomains identified by the displacement analysis (a-d). The switch domain was assigned to a "flexible" subdomain (grey color). Depending on the structures being compared the boundaries between subdomains change; e.g., KH2 is included in the plus domain in (f) and in the minus subdomain in (g) and (h). There are similarities between the KIF14 and Kinesin-1 identified subdomains. The KIF14 minus subdomain is similar to the kinesin-1 P-loop subdomain, and the KIF14 plus subdomain includes the kinesin-1 tubulin binding subdomain. However, the KIF14 switch subdomain includes only the switch 1 and switch 2 regions while the kinesin-1 switch I/II subdomain is larger and includes regions that are part of the KIF14 plus and minus subdomains. Differences between the KIF14 and kinesin-1 subdomain boundaries arise from the distinct structures being compared and the inherent limitation of applying subdomain finding algorithms to structures without rigid subdomains. Thus, any of these kinesin motor subdomain definitions should be considered an approximation to describe regions within the kinesin motor domain that tend to move as a unit relative to each other but without narrowly defined boundaries or hinges between them. A distinction between the kinesin-1 and the KIF14 proposed subdomains is that rather than a single 'tubulin binding' subdomain, in the case of KIF14 the kinesin-microtubule interface is formed by the three subdomains (supplementary Fig. 8).

a

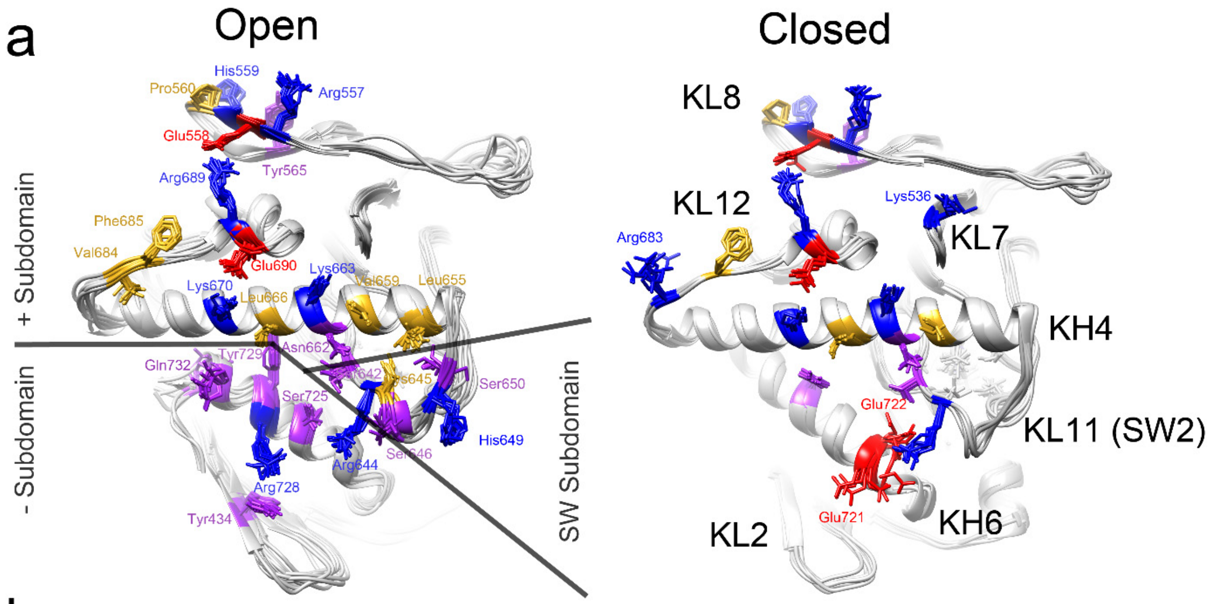


b

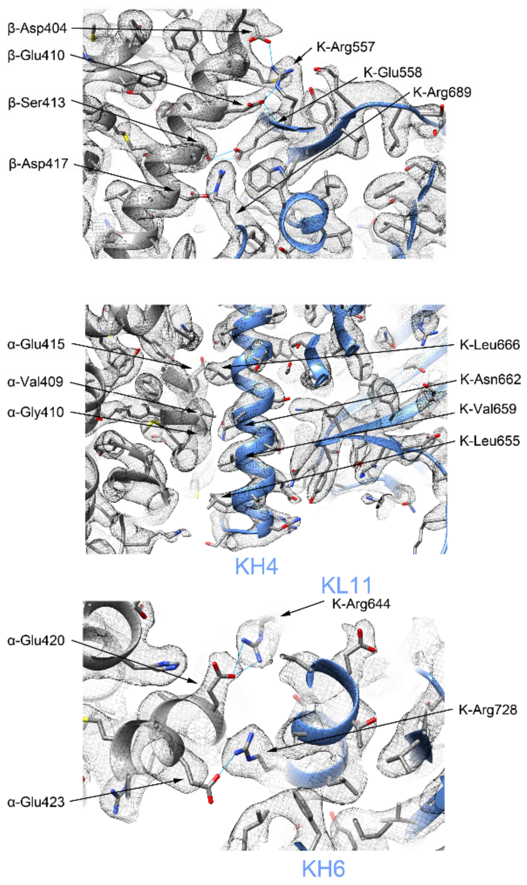


c

| KIF14 res | Tub res | Kin region | open_freq | closed_freq |
|---|---|---|---|---|
| K-TYR434 | A-GLU423 | L2 | 0.50 | 0.14 |
| K-LYS536 | B-GLU157 | B5 | 0.36 | 0.86 |
| K-ARG557 | B-GLU410 | L8 | 1.00 | 1.00 |
| K-ARG557 | B-MET406 | L8 | 1.00 | 1.00 |
| K-GLU558 | B-MET406 | L8 | 0.71 | 0.71 |
| K-GLU558 | B-GLU410 | L8 | 0.86 | 1.00 |
| K-GLU558 | B-SER413 | L8 | 0.79 | 0.71 |
| K-HIS559 | B-MET406 | L8 | 0.57 | 0.57 |
| K-PRO560 | B-THR409 | L8 | 0.79 | 0.71 |
| K-TYR565 | B-MET406 | L8 | 1.00 | 0.86 |
| K-SER642 | A-GLU414 | L11(SW2) | 1.00 | 0.71 |
| K-ARG644 | A-GLU417 | L11(SW2) | 0.71 | 0.71 |
| K-ARG644 | A-GLU420 | L11(SW2) | 0.79 | 0.71 |
| K-ARG644 | A-GLU414 | L11(SW2) | 0.71 | 1.00 |
| K-CYS645 | A-TYR108 | L11(SW2) | 0.79 | 0.43 |
| K-CYS645 | A-GLY412 | L11(SW2) | 0.57 | 0.00 |
| K-SER646 | A-TYR108 | L11(SW2) | 0.93 | 0.00 |
| K-HIS649 | A-TYR108 | L11(SW2) | 0.64 | 0.14 |
| K-SER650 | A-TYR108 | L11(SW2) | 0.93 | 0.14 |
| K-LEU655 | A-TYR108 | H4 | 0.86 | 0.29 |
| K-VAL659 | A-GLY410 | H4 | 1.00 | 1.00 |
| K-VAL659 | A-VAL409 | H4 | 0.93 | 0.86 |
| K-VAL659 | A-GLY412 | H4 | 0.21 | 0.57 |
| K-ASN662 | A-VAL409 | H4 | 0.57 | 1.00 |
| K-LYS663 | A-VAL409 | H4 | 1.00 | 1.00 |
| K-LYS663 | A-GLY410 | H4 | 0.57 | 0.29 |
| K-LEU666 | A-GLU415 | H4 | 0.86 | 0.86 |
| K-LEU666 | A-VAL405 | H4 | 0.93 | 1.00 |
| K-LEU666 | A-ARG402 | H4 | 0.86 | 0.57 |
| K-LYS670 | A-ARG402 | H4 | 0.71 | 0.86 |
| K-ARG683 | B-ASP427 | L12 | 0.43 | 1.00 |
| K-VAL684 | B-GLN424 | L12 | 0.64 | 0.43 |
| K-PHE685 | B-GLN424 | L12 | 0.71 | 0.86 |
| K-PHE685 | B-GLU421 | L12 | 0.86 | 1.00 |
| K-PHE685 | B-SER420 | L12 | 0.57 | 0.71 |
| K-PHE685 | B-ASP417 | L12 | 0.57 | 0.57 |
| K-ARG689 | B-ASP417 | L12 | 1.00 | 1.00 |
| K-ARG689 | B-ARG262 | L12 | 0.71 | 0.86 |
| K-ARG689 | B-ASN414 | L12 | 1.00 | 0.86 |
| K-ARG689 | B-SER413 | L12 | 0.86 | 1.00 |
| K-GLU690 | B-PRO261 | L12 | 0.86 | 1.00 |
| K-GLU690 | B-ARG262 | L12 | 0.57 | 0.57 |
| K-GLU721 | A-GLU420 | H6 | 0.21 | 0.71 |
| K-GLU722 | A-GLU414 | H6 | 0.00 | 0.57 |
| K-SER725 | A-GLU414 | H6 | 0.71 | 0.00 |
| K-ARG728 | A-SER419 | H6 | 1.00 | 0.00 |
| K-ARG728 | A-GLU423 | H6 | 0.64 | 0.00 |
| K-TYR729 | A-GLU415 | H6 | 0.50 | 0.86 |
| K-TYR729 | A-ARG402 | H6 | 0.00 | 0.86 |
| K-GLN732 | A-ARG402 | H6 | 0.64 | 0.00 |

**Supplementary Fig. 8. KIF14-Microtubule interacting residues. (a)** KIF14 residue side chains identified as making contacts with the microtubule by the UCSF-Chimera routine find clashes/contacts. Residue side chains are colored by type (polar purple, hydrophobic yellow, negatively charged red and positively charged blue).

Lines indicate the boundaries between the three KIF14 motor subdomain regions (Supplementary Fig. 7). The left panel shows superimposed all the models in the open conformation and the right panel in the closed conformation. **(b)** Detailed view of three regions of the KIF14-microtubule interface. Structures displayed from top to bottom panel: MT-K772-AAF-L, MT-K772-AAF-T, MT-K772-AAF-L. KIF14 ribbons in blue, tubulin in gray and cryo-EM density as a semitransparent grey mesh. **(c)** List of identified contacts (K: KIF14, A: α-tubulin; B: β-tubulin). Kin region indicates the location of the given contact residue in the KIF14 motor domain (Sn: β-strand n, Ln: Loop n, Hn: α-helix n). open_freq and closed_freq indicate the frequency of model structures with the given contact in the open or closed conformations. Contacts between residues were found using UCSF-Chimera find contacts routine with default parameters. Only model structures with an overall resolution of 3.5 Å or better, where the positions of the side chains are well resolved, where used to identify the contacts. In (a) and (c) only contacts with frequencies >= 0.5 are shown.

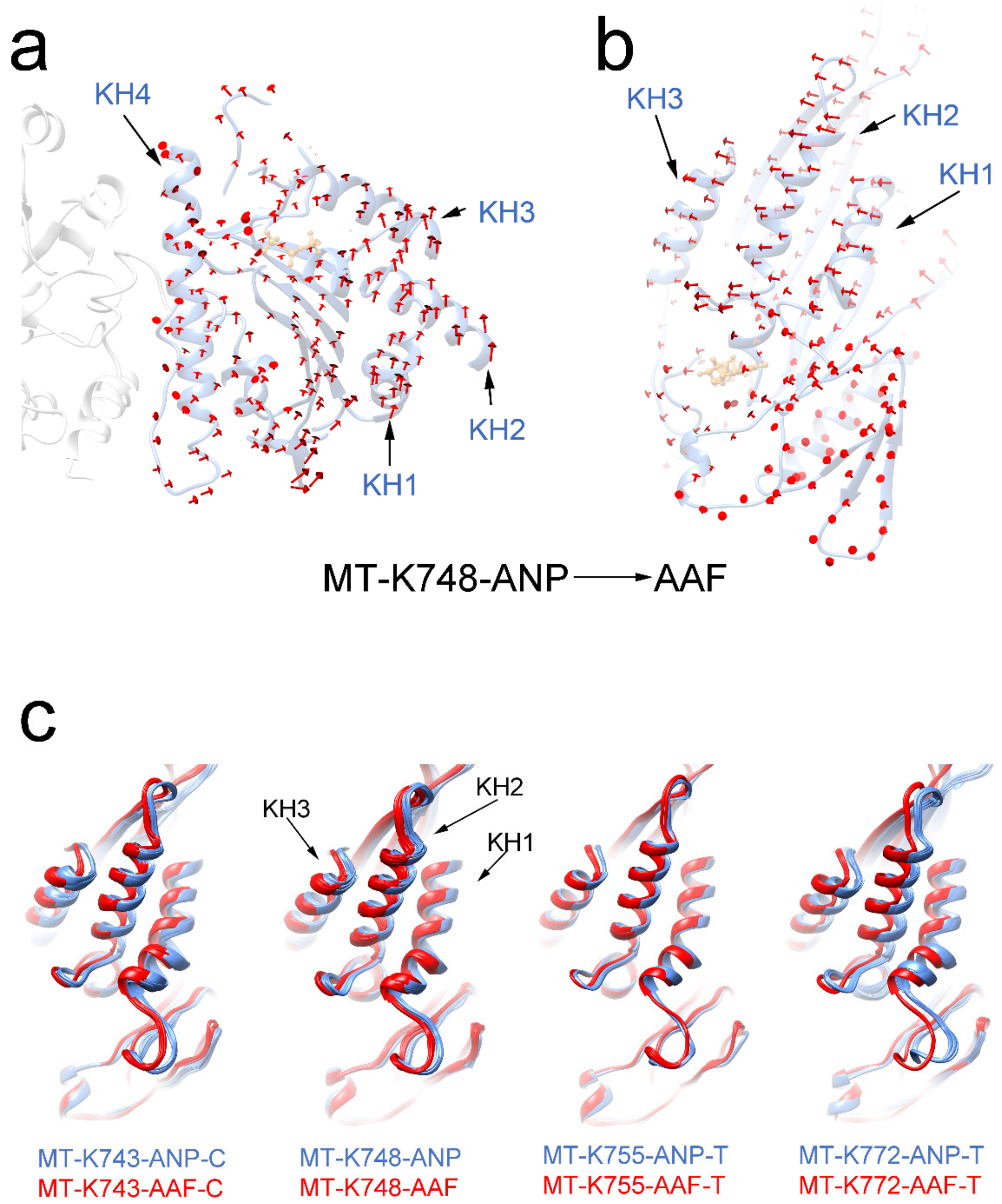


**Supplementary Fig. 9. Closed AMP-PNP vs. ADP-AlF$_x$ structures. (a-b)** MT-K748-ANP and MT-K748-AAF structure comparison. The figure shows a slab of the MT-K748-ANP structure with the red arrows indicating the displacement of Cα carbons between the MT-K48-AAF and MT-K748-ANP, after aligning the two complexes to the microtubule. View in (a) is from the microtubule minus end and in (b) towards the microtubule with the microtubule plus end at the top. As shown by the arrows the motor domain rotates relative to the microtubule from the ANP to the AAF complex structures (counterclockwise when viewed from the microtubule minus end). The displacement between the structures is higher at regions located further away from the microtubule, such as KH1, KH2 and KH3. **(c-d)** ANP (blue) vs. AAF (red) closed structures in four structure pairs (view as in (b)). For each structure and nucleotide condition the twenty Rosetta fitted models used to estimate coordinate precision (Supplementary Fig. 5) are superimposed. All structures aligned to the microtubule as in (a) and (b). Note that in the four structure pairs KH1, KH2 and KH3 are displaced to the left in the AAF relative to the ANP complexes. Note also that the displacement is higher than the variability between corresponding precision estimation model groups.

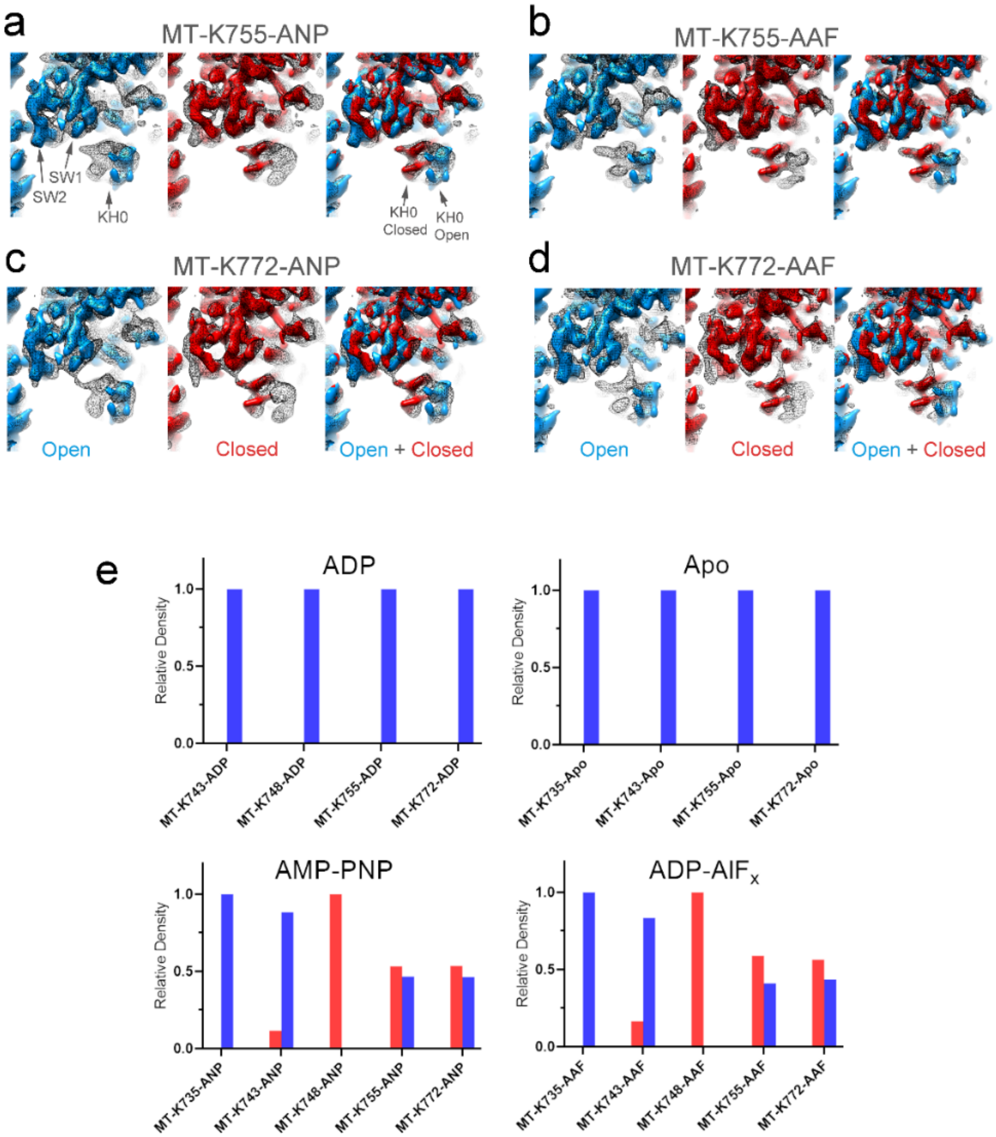


**Supplementary Fig. 10. Coexisting conformations in helically-averaged cryo-EM maps. (a-d)** Each panel shows a region of the cryo-EM densities of the helically-averaged map (iso-density semitransparent grey surface mesh) of the four complex structures indicated in the label. For comparison, the cryo-EM maps of complexes in which the KIF14 motor domain is in the open (MT-K748-ADP, blue iso-density surface) or closed configuration (MT-K748-ANP, red iso-density surface) are superimposed. All maps are aligned to their corresponding microtubule β- tubulin structures. Note that the cryo-EM maps of the MT-K755 and MT-K772 ANP or AAF complex contains a combination of the open and closed cryo-EM densities. Note also that in the areas where secondary structures elements such as KH0 locate in two distinct positions in the open and closed structures, the two corresponding densities are present and clearly resolved in the helically-averaged MT-K755 and MT-K772 maps. **(e)** Relative density at the two alternate locations (closed or open/open*) in helically-averaged cryo-EM map. Relative densities were calculated as $RD_x = D_x / (D_O + D_C)$, where Dx corresponds to the average density minus background at the open ($D_o$) or closed regions ($D_c$) (see methods). Note that in all the ADP and Apo structures the densities are associated with a single conformation (open). The MT-K748 associated densities are also associated with a single conformation, open or closed depending on nucleotide. On the other hand, the longer constructs K755 and K772 in the presence of AMP-PNP or ADP-$AlF_x$ present an almost equal mix of open and closed conformation associated densities. There is also a relatively smaller proportion of densities associated with the closed conformation in the MT-743-ANP and MT-743-AAF complexes.

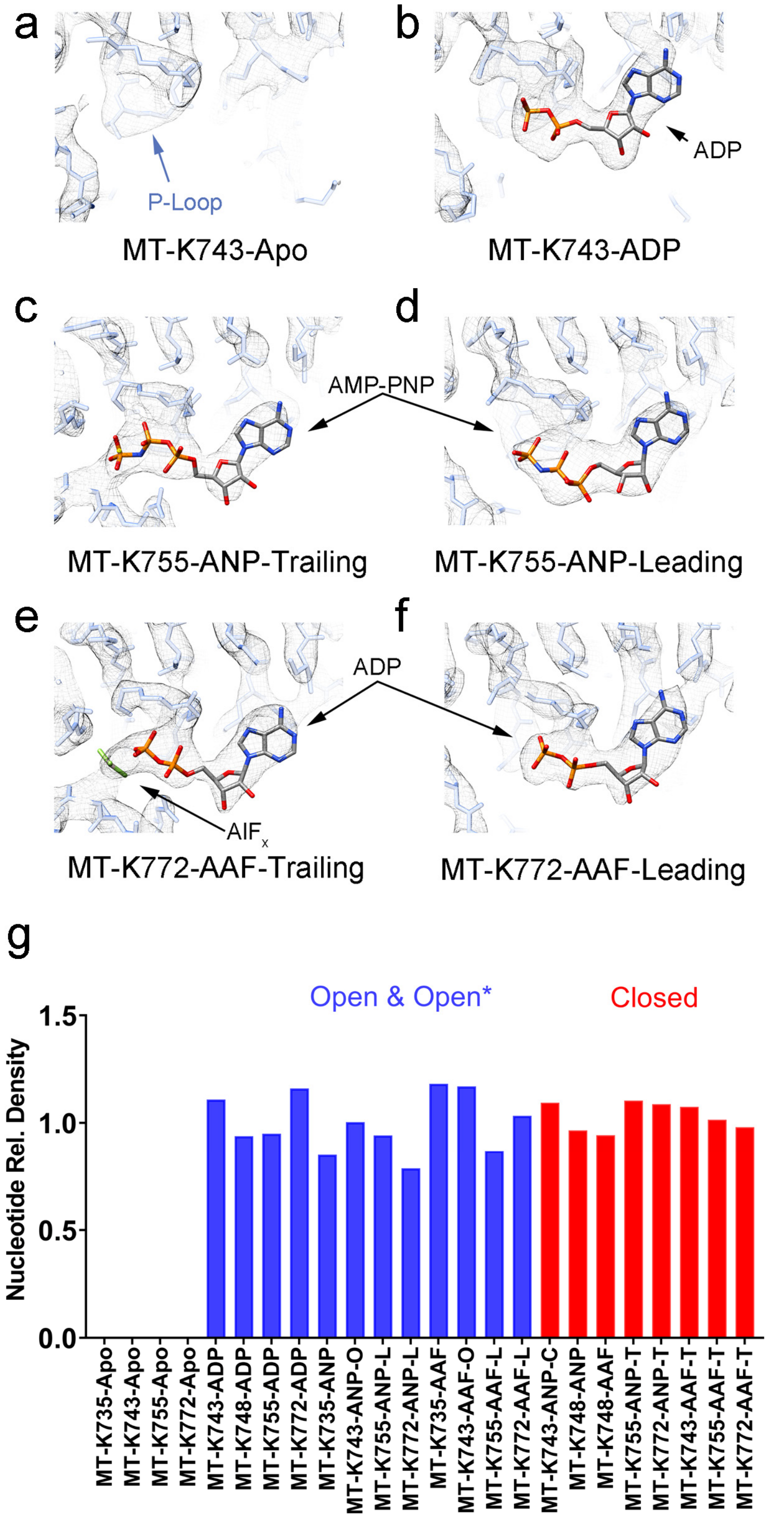


**Supplementary Fig. 11. Nucleotide associated densities in the KIF14 nucleotide binding pocket. (a-f)** Details of the nucleotide binding pocket of four complexes representing the four experimental nucleotide conditions used (Apo, ADP, ANP, and AAF). Structures in (a), (b), (d) and (f) corresponds to the open/open* conformations and to the closed conformation in (c) and (e). **(g)** Relative density at the nucleotide base ring position of the nucleotide binding pocket of all cryo-EM maps. Relative nucleotide density (RND) was calculated as RND=ND/PLD where ND and PLD are the average density minus background at the nucleotide base and P-loop locations respectively (see methods).

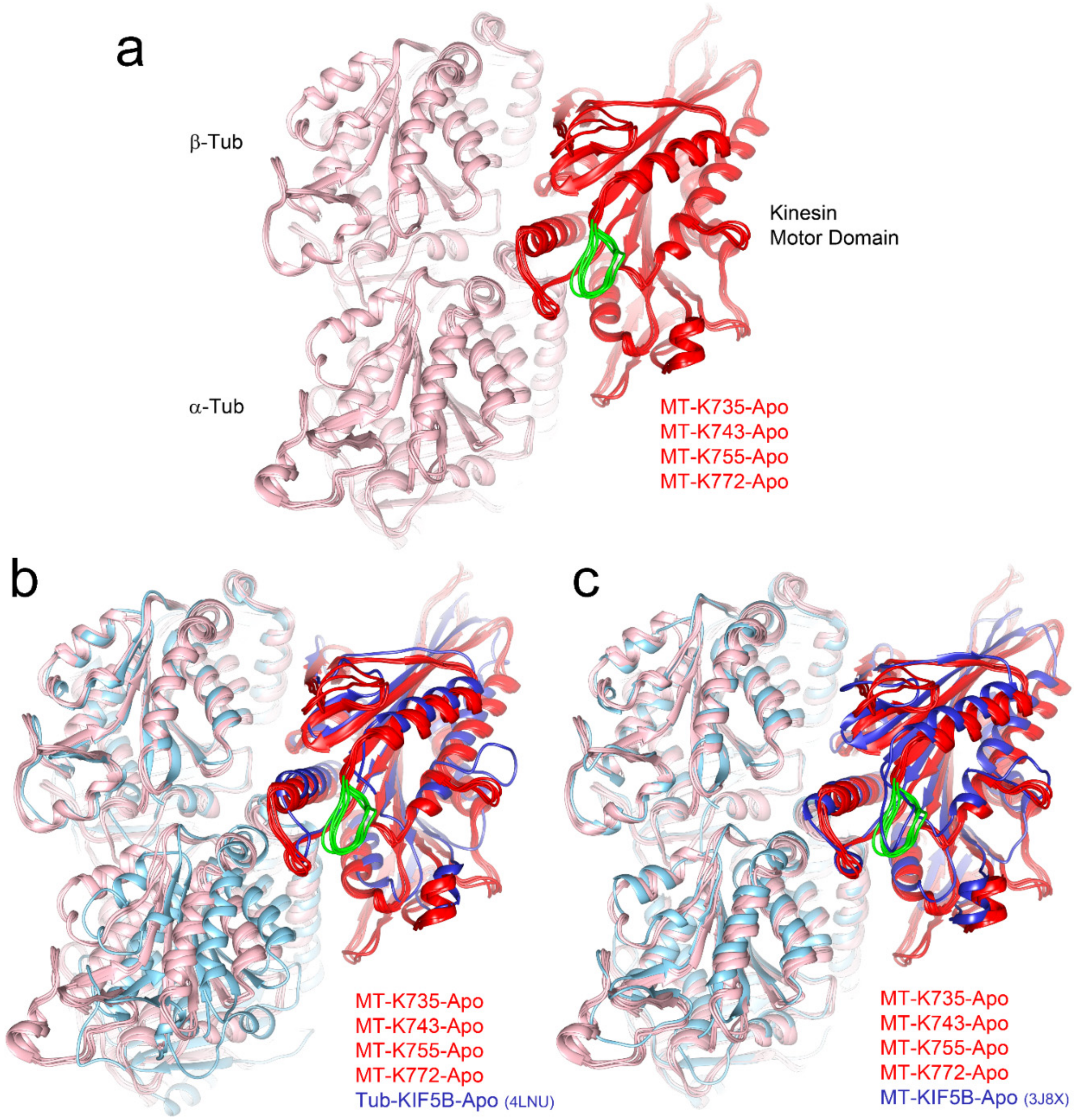


**Supplementary Fig. 12. KIF14 and kinesin-1 microtubule/tubulin complexes comparison. (a)** Four microtubule KIF14 apo complexes (MT-K735-Apo, MT-K743-Apo, MT-K755-Apo, MT-K772-Apo) superimposed by best alignment to their respective β-tubulin subunits. **(b)** Curved-tubulin-kinesin-1 (KIF5B) apo complex crystal structure (PDB: 4LNU) superimposed to the same structures in (a) using the same alignment. **(c)** Microtubule-KIF5B apo complex cryo-EM structure (PDB: 3J8X) superimposed to the same structures in (a) and using the same alignment. KIF14 complex structures are colored red and the KIF5B complexes in blue. Parts of the SW1 region of the KIF14 structures that are not resolved in the curved-tubulin-kinesin-1 apo complex are colored green. Note that although the structures of the kinesin motor domain in the Apo-KIF14 and Apo-KIFB complexes are similar (all in an 'open' conformation) their orientation and position relative to the bound tubulin subunit in the complexes are different.

## Supplementary References

1. Cao L, Wang W, Jiang Q, Wang C, Knossow M, Gigant B. The structure of apo-kinesin bound to tubulin links the nucleotide cycle to movement. *Nature Communications* **5**, 5364- (2014).

2. Mosca R, Schneider TR. RAPIDO: a web server for the alignment of protein structures in the presence of conformational changes. *Nucleic Acids Research* **36**, W42-W46 (2008).
