# Supplementary material for: Structural basis of mechano-chemical coupling by the mitotic kinesin KIF14
Source: Nat Commun. 2021 Jun 15;12:3637. doi: 10.1038/s41467-021-23581-3 (PMC8206134; doi:10.1038/s41467-021-23581-3)
Supplement: Supplementary file 3 — Description of Additional Supplementary Files [file 41467_2021_23581_MOESM3_ESM.docx]

**Description of Additional Supplementary Files**

File Name: **Supplementary Movie 1. Major conformations of the KIF14-microtubule complex through the ATPase cycle**.

Description: Movie made from seven structural models of KIF14 monomeric constructs, one from the microtubule unbound KIF14-ADP structure (4OZQ) and six from the microtubule-bound complexes (MT-748-ADP, MT-743-Apo, MT-743-ANP-O, MT-748-ANP, MT-748-AAF, MT-743-AAF-O, MT-748-ADP). Structural transitions between states estimated by linear interpolation. Four successive different angles of view are displayed. The models were aligned on their α- and β-tubulin subunits. β- and α-tubulin are colored in dark and light gray respectively. The KIF14 motor is colored according to the particular conformational change of the structure, semi-open in green, open in cyan, open* in blue and closed in red. Color saturation adjusted according to the amount of displacement between the open and closed structures (larger displacement more saturation). The side chains of key KIF14 nucleotide pocket residues, Arg‑401, Pro-402, Tyr-490, Ser-489, Ser-603 and Asn-599, are displayed as yellow sticks. The coordinated magnesium ion is displayed as a yellow sphere. The nucleotide is displayed in orange as sticks and the phosphate groups as spheres. The neck-linker (only visible in the closed structures) is colored in red. Loop-10 residues 618 to 627 are not displayed.

File Name: **Supplementary Movie 2. Kinesin dimer coordinated mechano-chemical cycle model.**

Description: Movie made from structural models of the MT-K755-ANP and MT-K755-AAF two-head bound states, and the microtubule unbound KIF14-ADP structure (4OZQ). Two successive angles of view are displayed. Structure representation and color scheme is as in Supplementary Movie 1. In the docked configuration the neck-linker and coiled coil are colored in red while in the undocked configuration they are colored in pink. The relative time between states is only illustrative.

File Name: **Supplementary Data File.**

Description: Zip data folder containing the figures and tables source data files.
