# Supplementary material for: Structural basis of mechano-chemical coupling by the mitotic kinesin KIF14
Source: Nat Commun. 2021 Jun 15;12:3637. doi: 10.1038/s41467-021-23581-3 (PMC8206134; doi:10.1038/s41467-021-23581-3)
Supplement: Supplementary file 6 — Reporting Summary [file 41467_2021_23581_MOESM6_ESM.pdf]

nature research

Corresponding author(s): Hernando Sosa, Benjamin Kwok

Last updated by author(s): 2021/04/26

Reporting Summary

Nature Research wishes to improve the reproducibility of the work that we publish. This form provides structure for consistency and transparency in reporting. For further information on Nature Research policies, see our Editorial Policies and the Editorial Policy Checklist.

Statistics

For all statistical analyses, confirm that the following items are present in the figure legend, table legend, main text, or Methods section.

n/a	Confirmed	
☐	☒	The exact sample size (n) for each experimental group/condition, given as a discrete number and unit of measurement
☐	☒	A statement on whether measurements were taken from distinct samples or whether the same sample was measured repeatedly
☒	☐	The statistical test(s) used AND whether they are one- or two-sided<br>*Only common tests should be described solely by name; describe more complex techniques in the Methods section.*
☒	☐	A description of all covariates tested
☒	☐	A description of any assumptions or corrections, such as tests of normality and adjustment for multiple comparisons
☒	☐	A full description of the statistical parameters including central tendency (e.g. means) or other basic estimates (e.g. regression coefficient) AND variation (e.g. standard deviation) or associated estimates of uncertainty (e.g. confidence intervals)
☒	☐	For null hypothesis testing, the test statistic (e.g. F, t, r) with confidence intervals, effect sizes, degrees of freedom and P value noted<br>*Give P values as exact values whenever suitable.*
☒	☐	For Bayesian analysis, information on the choice of priors and Markov chain Monte Carlo settings
☒	☐	For hierarchical and complex designs, identification of the appropriate level for tests and full reporting of outcomes
☒	☐	Estimates of effect sizes (e.g. Cohen's d, Pearson's r), indicating how they were calculated

Our web collection on statistics for biologists contains articles on many of the points above.

Software and code

Policy information about availability of computer code

Data collection	Leginon (beta version)<br>UNICORN (v. 5.11)<br>TECAN i-control (3.9.1.0)
Data analysis	MotionCor 2 1.1.0<br>Gctf 1.06<br>spider 22.10<br>eman 1 1.9<br>eman 2 2.12<br>frealign 9.11<br>cistem 1.0.0-beta<br>relion 3.0 to 3.1<br>bsoft 1.9.5<br>localdeblur in xmipp 3.2 (in scipion 1.2.1)<br>modeller 9<br>rosetta 3 r59451<br>phenix 1.15.2 and 1.17<br>coot 0.8.9<br>UCSF-Chimera 1.14<br>VMD 1.9.4<br>R 3.1.1 and 3.3<br>Python 2.7.14

Microsoft Excel 2007
Prism-GraphPad 7.04

For manuscripts utilizing custom algorithms or software that are central to the research but not yet described in published literature, software must be made available to editors and reviewers. We strongly encourage code deposition in a community repository (e.g. GitHub). See the Nature Research guidelines for submitting code & software for further information.

Data

Policy information about availability of data

All manuscripts must include a data availability statement. This statement should provide the following information, where applicable:

- Accession codes, unique identifiers, or web links for publicly available datasets
- A list of figures that have associated raw data
- A description of any restrictions on data availability

The datasets generated and/or analyzed during the current study are available from the corresponding author on reasonable request. Source data for figures and tables are included in supplementary file DataSource.zip. Atomic coordinates and corresponding cryo-EM density maps (HASRC refined maps and helically-averaged maps), including the half maps, masks and FSC curves used to estimate spatial resolution have been deposited in the Protein Data Bank (PDB) and Electron Microscopy Data Resource (EMD) under the accession codes 6WWE, 6WWF, 6WWG¸ 6WWH¸ 6WWI¸ 6WWJ¸ 6WWK¸ 6WWL¸6WWM¸ 6WWN¸ 6WWO¸ 6WWP¸ 6WWQ¸ 6WWR¸ 6WWS¸ 6WWT¸ 6WWU¸ 6WWV¸ 7LVQ, 7LVR and EMD-21932, EMD-21933, EMD-21934, EMD-21935, EMD-21936, EMD-21937, EMD-21938, EMD-21939, EMD-21940, EMD-21941, EMD-21942, EMD-21943, EMD-21944, EMD-21945, EMD-21946, EMD-21947, EMD-21948, EMD-21949, EMD-23540, EMD-23541 (Table 1). They are accessible through the following hyperlinks:
https://www.rcsb.org/structure/6WWE
https://www.rcsb.org/structure/6WWF
https://www.rcsb.org/structure/6WWG
https://www.rcsb.org/structure/6WWH
https://www.rcsb.org/structure/6WWI
https://www.rcsb.org/structure/6WWJ
https://www.rcsb.org/structure/6WWK
https://www.rcsb.org/structure/6WWL
https://www.rcsb.org/structure/6WWM
https://www.rcsb.org/structure/6WWN
https://www.rcsb.org/structure/6WWO
https://www.rcsb.org/structure/6WWP
https://www.rcsb.org/structure/6WWQ
https://www.rcsb.org/structure/6WWR
https://www.rcsb.org/structure/6WWS
https://www.rcsb.org/structure/6WWT
https://www.rcsb.org/structure/6WWU
https://www.rcsb.org/structure/6WWV
https://www.rcsb.org/structure/7LVQ
https://www.rcsb.org/structure/7LVR
https://www.emdataresource.org/EMD-21932
https://www.emdataresource.org/EMD-21933
https://www.emdataresource.org/EMD-21934
https://www.emdataresource.org/EMD-21935
https://www.emdataresource.org/EMD-21936
https://www.emdataresource.org/EMD-21937
https://www.emdataresource.org/EMD-21938
https://www.emdataresource.org/EMD-21939
https://www.emdataresource.org/EMD-21940
https://www.emdataresource.org/EMD-21941
https://www.emdataresource.org/EMD-21942
https://www.emdataresource.org/EMD-21943
https://www.emdataresource.org/EMD-21944
https://www.emdataresource.org/EMD-21945
https://www.emdataresource.org/EMD-21946
https://www.emdataresource.org/EMD-21947
https://www.emdataresource.org/EMD-21948
https://www.emdataresource.org/EMD-21949
https://www.emdataresource.org/EMD-23540
https://www.emdataresource.org/EMD-23541

Field-specific reporting

Please select the one below that is the best fit for your research. If you are not sure, read the appropriate sections before making your selection.

☒ Life sciences ☐ Behavioural & social sciences ☐ Ecological, evolutionary & environmental sciences

For a reference copy of the document with all sections, see nature.com/documents/nr-reporting-summary-flat.pdf

Life sciences study design

All studies must disclose on these points even when the disclosure is negative.

Sample size	To produce 3D Cryo-EM maps at the highest possible resolution (near atomic) thousands of images were collected for each experimental condition as indicated in Table 1.
Data exclusions	Images were selected in a non-biased manner by image analysis software using well defined criteria (quality, microtubule type etc.). The number of particles identified as the selected microtubule type (15R) and the ones included in the final 3D reconstructions are indicated in Table 1.
Replication	ATPase measurements were done in triplicate (three independent experiments per protein prep).
Randomization	N/A
Blinding	N/A

Reporting for specific materials, systems and methods

We require information from authors about some types of materials, experimental systems and methods used in many studies. Here, indicate whether each material, system or method listed is relevant to your study. If you are not sure if a list item applies to your research, read the appropriate section before selecting a response.

Materials & experimental systems

n/a	Involved in the study	
☒	☐	Antibodies
☒	☐	Eukaryotic cell lines
☒	☐	Palaeontology and archaeology
☒	☐	Animals and other organisms
☒	☐	Human research participants
☒	☐	Clinical data
☒	☐	Dual use research of concern

Methods

n/a	Involved in the study	
☒	☐	ChIP-seq
☒	☐	Flow cytometry
☒	☐	MRI-based neuroimaging
