# Supplementary material for: Structural basis of mechano-chemical coupling by the mitotic kinesin KIF14
Source: Nat Commun. 2021 Jun 15;12:3637. doi: 10.1038/s41467-021-23581-3 (PMC8206134; doi:10.1038/s41467-021-23581-3)
Supplement: Supplementary file 7 — Source Data [file 41467_2021_23581_MOESM7_ESM.zip › DataSource/Supplementary_Fig1_data.docx]

**391**

*****

mmKIF14 344 ASPRVKPRPKSSLFANKRESSRESTLPPEENSLVQKTFTEPDSLKVENSQVTVAVRVRPF
hsKIF14 309 SNLQVKQRPKSSFLANKQERSAENTILPEEETVVQNTSAGKDPLKVENSQVTVAVRVRPF

 **____________________________________________________________**
mmKIF14 404 SKREKTEKASQVVFTNGEEITVEHPDMKQVYSFIYDVSFWSFDECHPGYASQTTVYETLA
hsKIF14 369 TKREKIEKASQVVFMSGKEITVEHPDTKQVYNFIYDVSFWSFDECHPHYASQTTVYEKLA

 **____________________________________________________________**
mmKIF14 464 APLLDRAFEGYNTCLFAYGQTGSGKSYTMMGLNEEPGIIPRFCEDLFAQIAKKQTSEVSY
hsKIF14 429 APLLERAFEGFNTCLFAYGQTGSGKSYTMMGFSEEPGIIPRFCEDLFSQVARKQTQEVSY

 **____________________________________________________________**
mmKIF14 524 HLEMSFFEVYNEKIHDLLVCKGENGQRKQPLRAREHPVSGPYVEGLSMNVVSSYSDIQSW
hsKIF14 489 HIEMSFFEVYNEKIHDLLVCKDENGQRKQPLRVREHPVYGPYVEALSMNIVSSYADIQSW

 **____________________________________________________________**
mmKIF14 584 LELGNKQRATAATGMNDKSSRSHSVFTLVMTQTKTEVVEGEEHDHRITSRINLVDLAGSE
hsKIF14 549 LELGNKQRATAATGMNDKSSRSHSVFTLVMTQTKTEFVEGEEHDHRITSRINLIDLAGSE

 **____________________________________________________________**
mmKIF14 644 RCSTAHSSGQRLKEGVSINKSLLTLGKVISALSEQANGKRVFIPYRESTLTWLLKESLGG
hsKIF14 609 RCSTAHTNGDRLKEGVSINKSLLTLGKVISALSEQANQRSVFIPYRESVLTWLLKESLGG

 **735 743 748 755**

**_______________________________*_______*____* *________**
mmKIF14 704 NSKTAMIATVSPAASNIEETLSTLRYATQARLIVNIAKVNEDMNAKLIRELKAEIEKLKA
hsKIF14 669 NSKTAMIATISPAASNIEETLSTLRYANQARLIVNIAKVNEDMNAKLIRELKAEIAKLKA

**772**
 **________***
mmKIF14 764 AQRSNRNIDPERYRLCRQEITSLRMKLHQQERDMAEIQRVWKEKFEQAEKRKLQETKELQ
hsKIF14 729 AQRNSRNIDPERYRLCRQEITSLRMKLHQQERDMAEMQRVWKEKFEQAEKRKLQETKELQ
