# Supplementary material for: Structural basis of mechano-chemical coupling by the mitotic kinesin KIF14
Source: Nat Commun. 2021 Jun 15;12:3637. doi: 10.1038/s41467-021-23581-3 (PMC8206134; doi:10.1038/s41467-021-23581-3)
Supplement: Supplementary file 7 — Source Data [file 41467_2021_23581_MOESM7_ESM.zip › DataSource/Table1_helicalAndLocal_v15.docx]

**Table 1. Cryo-EM data collection, refinement and validation statistics (1/5)**

|  | K735-ANP  (EMDB-21949)  (PDB 6WWV) | K735-AAF  (EMDB-21948)  (PDB 6WWU) | K735 Apo  (EMDB-21947)  (PDB 6WWT) | K743-ANP  open state class  (EMDB-21946)  (PDB 6WWS) |
| --- | --- | --- | --- | --- |
| **Data collection and processing** |  |  |  |  |
| Magnification (actual) | 46598 | 60168 | 46598 | 58893 |
| Voltage (kV) | 300 | 300 | 300 | 300 |
| Electron exposure (e^–^/Å^2^) | 69.9 | 70.1 | 69.9 | 63.0 |
| Defocus range (μm) ^a^ | 1.0 - 1.9 | 1.1 - 1.9 | 1.0 - 2.0 | 0.9 - 1.6 |
| Pixel size (Å) | 1.073 | 0.831 | 1.073 | 0.849 |
| Symmetry imposed ^b^  Rise (Å)  Twist (deg) | Helical  5.5  168.09 | Helical  5.41  168.07 | Helical  5.46  168.09 | Helical  5.45  168.09 |
| Particle images identified as 15R symmetry (no.) | 15350 | 18849 | 9865 | 38121 |
| Particle images in helical reconstruction (no.) | 15350 | 18849 | 9865 | 38121 |
| Single particles (no.) ^c^ | 230250 | 282735 | 147975 | 571815 |
| Single particles used (no.) ^d^ | 139592 | 217725 | 101569 | 324809 |
| Overall resolution (Å)  FSC threshold | 3.1  0.143 | 2.7  0.143 | 3.2  0.143 | 2.7  0.143 |
| Kinesin resolution (Å) | 3.2 | 2.9 | 3.3 | 2.8 |
| Tubulin resolution (Å) | 3.0 | 2.7 | 3.2 | 2.6 |
| Helical resolution (Å) ^e^ | 3.5 | 3.0 | 3.6 | 3.0 |
|  |  |  |  |  |
| **Refinement** |  |  |  |  |
| Initial model used (PDB code) ^f^ | 6B0I | 6B0I | 6B0I | 6B0I |
| Model composition  Non-hydrogen atoms  Protein residues  Ligands | 9676  1215  5 | 9688  1216  5 | 9657  1215  4 | 9697  1216  5 |
| R.m.s. deviations  Bond lengths (Å)  Bond angles (°) | 0.008  1.21 | 0.0101  1.42 | 0.0083  1.43 | 0.0090  1.42 |
| Validation  MolProbity score  Clashscore  Poor rotamers (%) | 1.74  5.67  0.67 | 2.12  6.82  2.78 | 1.92  6.00  1.53 | 2.25  8.07  3.06 |
| Ramachandran plot  Favored (%)  Allowed (%)  Disallowed (%) | 93.47  5.96  0.58 | 93.97  5.79  0.25 | 92.89  6.78  0.33 | 93.14  6.53  0.33 |

Notes:

^a^ Range of the average defocus measured on the particle images used for the helical reconstructions. The range comprises 90% of the particles used (5% of the particle defocuses are below and 5% are above this range).

^b^ Symmetry imposed on the helically aveaged map only.

^c^ Total number of particles after symmetry expansion.

^d^ Number of single particles used after 3D classification. For single-head-bound states these single particles correspond to a kinesin motor bound to a tubulin dimer, and for two-head-bound kinesin states (755-ANP, 755-AAF, 772-ANP, and 772-AAF) they correspond to two connected kinesin motors bound to two tubulin dimers (i.e. half the corresponding amount of particles from symmetry expansion).

^e^ Overall resolution of the helically averaged map.

^f^ 15R decorated microtubule model used as the start of cryo-EM processing.

**Table 1. continued (2/5)**

|  | K743-ANP  closed state class  (EMDB-23540)  (PDB 7LVQ) | K743-AAF  open state class  (EMDB-21945)  (PDB 6WWR) | K743-AAF closed state class  (EMDB-23541)  (PDB 7LVR) | K743-ADP  (EMDB-21944)  (PDB 6WWQ) |
| --- | --- | --- | --- | --- |
| **Data collection and processing** |  |  |  |  |
| Magnification (actual) | 58893 | 60606 | 60606 | 60386 |
| Voltage (kV) | 300 | 300 | 300 | 300 |
| Electron exposure (e^–^/Å^2^) | 63.0 | 70.8 | 70.8 | 70.2 |
| Defocus range (μm) ^a^ | 0.9 - 1.6 | 0.8 - 1.7 | 0.8 - 1.7 | 0.8 - 1.6 |
| Pixel size (Å) | 0.849 | 0.825 | 0.825 | 0.828 |
| Symmetry imposed ^b^  Rise (Å)  Twist (deg) | Helical  5.45  168.09 | Helical  5.44  168.07 | Helical  5.44  168.07 | Helical  5.42  168.08 |
| Particle images identified as 15R symmetry (no.) | 38121 | 41714 | 41714 | 5194 |
| Particle images in helical reconstruction (no.) | 38121 | 41714 | 41714 | 5194 |
| Single particles (no.) ^c^ | 571815 | 625710 | 625710 | 77910 |
| Single particles used (no.) ^d^ | 136802 | 348209 | 162897 | 61697 |
| Overall resolution (Å)  FSC threshold | 2.9  0.143 | 2.7  0.143 | 2.9  0.143 | 3.0  0.143 |
| Kinesin resolution (Å) | 3.1 | 2.9 | 3.1 | 3.3 |
| Tubulin resolution (Å) | 2.8 | 2.6 | 2.7 | 3.0 |
| Helical resolution (Å) ^e^ | 3.0 | 3.0 | 3.0 | 3.3 |
|  |  |  |  |  |
| **Refinement** |  |  |  |  |
| Initial model used (PDB code) ^f^ | 6B0I | 6B0I | 6B0I | 6B0I |
| Model composition  Non-hydrogen atoms  Protein residues  Ligands | 9768  1226  6 | 9719  1220  5 | 9802  1231  7 | 9675  1215  5 |
| R.m.s. deviations  Bond lengths (Å)  Bond angles (°) | 0.0237  1.48 | 0.0089  1.19 | 0.0126  1.25 | 0.0087  1.19 |
| Validation  MolProbity score  Clashscore  Poor rotamers (%) | 1.71  5.31  0.66 | 1.68  5.29  0.57 | 1.53  3.79  0.19 | 1.67  5.15  0.19 |
| Ramachandran plot  Favored (%)  Allowed (%)  Disallowed (%) | 93.52  6.39  0.08 | 94.15  5.85  0.00 | 94.78  5.22  0.00 | 94.13  5.87  0.00 |

**Table 1. continued (3/5)**

|  | K743 Apo  (EMDB-21943)  (PDB 6WWP) | K748-ANP  (EMDB-21942)  (PDB 6WWO) | K748-AAF  (EMDB-21941)  (PDB 6WWN) | K748-ADP  (EMDB-21940)  (PDB 6WWM) |
| --- | --- | --- | --- | --- |
| **Data collection and processing** |  |  |  |  |
| Magnification (actual) | 58962 | 60386 | 60606 | 58893 |
| Voltage (kV) | 300 | 300 | 300 | 300 |
| Electron exposure (e^–^/Å^2^) | 70.0 | 68.2 | 70.5 | 62.4 |
| Defocus range (μm) ^a^ | 0.9 - 1.8 | 0.9 - 1.7 | 1.0 - 1.7 | 0.8 - 1.9 |
| Pixel size (Å) | 0.848 | 0.828 | 0.825 | 0.849 |
| Symmetry imposed ^b^  Rise (Å)  Twist (deg) | Helical  5.45  168.09 | Helical  5.43  168.08 | Helical  5.41  168.07 | Helical  5.51  168.09 |
| Particle images identified as 15R symmetry (no.) | 12644 | 16440 | 15178 | 14102 |
| Particle images in helical reconstruction (no.) | 12644 | 11075 | 15178 | 14102 |
| Single particles (no.) ^c^ | 189660 | 246600 | 227670 | 211530 |
| Single particles used (no.) ^d^ | 147872 | 166809 | 161836 | 111394 |
| Overall resolution (Å)  FSC threshold | 3.1  0.143 | 2.8  0.143 | 3.5  0.143 | 2.8  0.143 |
| Kinesin resolution (Å) | 3.3 | 2.9 | 3.5 | 3.0 |
| Tubulin resolution (Å) | 3.0 | 2.8 | 3.4 | 2.7 |
| Helical resolution (Å) ^e^ | 3.3 | 3.2 | 3.9 | 3.1 |
|  |  |  |  |  |
| **Refinement** |  |  |  |  |
| Initial model used (PDB code) ^f^ | 6B0I | 6B0I | 6B0I | 6B0I |
| Model composition  Non-hydrogen atoms  Protein residues  Ligands | 9646  1213  4 | 9784  1230  6 | 9784  1230  7 | 9665  1212  5 |
| R.m.s. deviations  Bond lengths (Å)  Bond angles (°) | 0.0068  1.18 | 0.0102  1.41 | 0.0068  1.20 | 0.0118  1.24 |
| Validation  MolProbity score  Clashscore  Poor rotamers (%) | 1.80  6.69  0.67 | 1.98  5.92  1.99 | 1.78  6.13  0.66 | 1.73  5.42  0.77 |
| Ramachandran plot  Favored (%)  Allowed (%)  Disallowed (%) | 93.29  6.55  0.17 | 93.55  6.13  0.33 | 93.14  6.70  0.16 | 93.12  6.88  0.00 |

**Table 1. continued (4/5)**

|  | K755 ANP  (EMDB-21939)  (PDB 6WWL) | K755-AAF  (EMDB-21938)  (PDB 6WWK) | K755-ADP  (EMDB-21937)  (PDB 6WWJ) | K755 Apo  (EMDB-21936)  (PDB 6WWI) |
| --- | --- | --- | --- | --- |
| **Data collection and processing** |  |  |  |  |
| Magnification (actual) | 46624 | 58962 | 60168 | 45956 |
| Voltage (kV) | 300 | 300 | 300 | 300 |
| Electron exposure (e^–^/Å^2^) | 72.1 | 73.4 | 70.6 | 69.2 |
| Defocus range (μm) ^a^ | 0.6 - 2.0 | 0.6 - 1.7 | 0.7 - 1.7 | 0.7 - 1.8 |
| Pixel size (Å) | 1.0724 | 0.848 | 0.831 | 1.088 |
| Symmetry imposed ^b^  Rise (Å)  Twist (deg) | Helical  5.47  168.09 | Helical  5.45  168.07 | Helical  5.46  168.09 | Helical  5.5  169.09 |
| Particle images identified as 15R symmetry (no.) | 32006 | 26000 | 18744 | 15798 |
| Particle images in helical reconstruction (no.) | 32006 | 26000 | 18744 | 15798 |
| Single particles (no.) ^c^ | 480090 | 390000 | 281160 | 236970 |
| Single particles used (no.) ^d^ | 152330 | 146982 | 171108 | 141605 |
| Overall resolution (Å)  FSC threshold | 3.1  0.143 | 3.0  0.143 | 3.4  0.143 | 3.6  0.143 |
| Kinesin resolution (Å) | 3.3 | 3.1 | 3.5 | 3.8 |
| Tubulin resolution (Å) | 3.1 | 2.9 | 3.3 | 3.5 |
| Helical resolution (Å) ^e^ | 3.4 | 3.2 | 3.5 | 3.8 |
|  |  |  |  |  |
| **Refinement** |  |  |  |  |
| Initial model used (PDB code) ^f^ | 6B0I | 6B0I | 6B0I | 6B0I |
| Model composition  Non-hydrogen atoms  Protein residues  Ligands | 19665  2470  11 | 19698  2473  11 | 9798  1230  5 | 9753  1229  4 |
| R.m.s. deviations  Bond lengths (Å)  Bond angles (°) | 0.0072  1.10 | 0.0073  1.10 | 0.0086  1.23 | 0.0110  1.25 |
| Validation  MolProbity score  Clashscore  Poor rotamers (%) | 1.49  4.88  0.94 | 1.71  4.79  1.65 | 1.80  6.69  0.57 | 1.84  6.71  0.47 |
| Ramachandran plot  Favored (%)  Allowed (%)  Disallowed (%) | 96.42  3.58  0.00 | 95.77  4.14  0.08 | 93.38  6.62  0.00 | 92.56  7.36  0.08 |

**Table 1. continued (5/5)**

|  | K772ANP  (EMDB-21935)  (PDB 6WWH) | K772-AAF  (EMDB-21934)  (PDB 6WWG) | K772-ADP  (EMDB-21933)  (PDB 6WWF) | K772 Apo  (EMDB-21932)  (PDB 6WWE) |
| --- | --- | --- | --- | --- |
| **Data collection and processing** |  |  |  |  |
| Magnification (actual) | 46598 | 60533 | 58893 | 58824 |
| Voltage (kV) | 300 | 300 | 300 | 300 |
| Electron exposure (e^–^/Å^2^) | 67.3 | 75.1 | 66.8 | 68.7 |
| Defocus range (μm) ^a^ | 0.8 - 2.0 | 0.7 - 1.6 | 0.5 - 1.5 | 0.7 – 1.9 |
| Pixel size (Å) | 1.073 | 0.826 | 0.849 | 0.850 |
| Symmetry imposed ^b^  Rise (Å)  Twist (deg) | Helical  5.48  168.09 | Helical  5.44  168.07 | Helical  5.48  168.09 | Helical  5.50  168.08 |
| Particle images identified as 15R symmetry (no.) | 3395 | 23479 | 7377 | 3843 |
| Particle images in helical reconstruction (no.) | 3395 | 23479 | 7377 | 3843 |
| Single particles (no.) ^c^ | 50925 | 352185 | 110385 | 57645 |
| Single particles used (no.) ^d^ | 21458 | 135651 | 73851 | 42831 |
| Overall resolution (Å)  FSC threshold | 3.8  0.143 | 2.9  0.143 | 3.3  0.143 | 3.9  0.143 |
| Kinesin resolution (Å) | 4.3 | 3.1 | 3.5 | 4.3 |
| Tubulin resolution (Å) | 3.6 | 2.8 | 3.2 | 3.8 |
| Helical resolution (Å) ^e^ | 3.9 | 3.0 | 3.5 | 3.9 |
|  |  |  |  |  |
| **Refinement** |  |  |  |  |
| Initial model used (PDB code) ^f^ | 6B0I | 6B0I | 6B0I | 6B0I |
| Model composition  Non-hydrogen atoms  Protein residues  Ligands | 19685  2471  12 | 19749  2479  12 | 9805  1231  5 | 9777  1231  4 |
| R.m.s. deviations  Bond lengths (Å)  Bond angles (°) | 0.0144  1.27 | 0.0068  1.16 | 0.0072  1.26 | 0.0069  1.23 |
| Validation  MolProbity score  Clashscore  Poor rotamers (%) | 2.35  11.82  2.92 | 2.01  6.48  2.58 | 1.66  4.97  0.47 | 1.84  7.11  0.19 |
| Ramachandran plot  Favored (%)  Allowed (%)  Disallowed (%) | 93.86  5.98  0.16 | 95.05  4.66  0.28 | 94.12  5.88  0.00 | 92.90  6.94  0.16 |
