# Supplementary material for: Structural basis of mechano-chemical coupling by the mitotic kinesin KIF14
Source: Nat Commun. 2021 Jun 15;12:3637. doi: 10.1038/s41467-021-23581-3 (PMC8206134; doi:10.1038/s41467-021-23581-3)
Supplement: Supplementary file 7 — Source Data [file 41467_2021_23581_MOESM7_ESM.zip › DataSource/Supplementary_Table_2.doc]

**Supplementary Table 2. Extra data collection and refinement parameters.**

| Dataset | Microscope  used | Pixel Size (Å) | Box size  helical  map (pixels) | Magnification anisotropy % ^a^ | Magnification anisotropy angle ^a^ | Per particle CTF refinement | 15R particle cleaning | Box size  (pixels) |
| --- | --- | --- | --- | --- | --- | --- | --- | --- |
| 735-ANP | Krios 1 | 1.073 | 512 | n.c. | n.c. | N | N | 320 |
| 735-AAF | Krios 1 | 0.831 | 664 | 0.33 | 88.7 | Y | N | 416 |
| 735 Apo | Krios 1 | 1.073 | 512 | n.c. | n.c. | N | N | 320 |
| 743-ANP | Krios 2 | 0.849 | 664 | 1.45 | 1 | Y | Y | 416 |
| 743-AAF | Krios 3 | 0.825 | 664 | 0.54 | 48.4 | Y | Y | 416 |
| 743-ADP | Krios 1 | 0.828 | 664 | 1.06 | 87.7 | Y | Y | 416 |
| 743 Apo | Krios 2 | 0.848 | 664 | 1.69 | 179 | Y | Y | 416 |
| 748-ANP | Krios 1 | 0.828 | 664 | 1.07 | 89.8 | Y | Y | 416 |
| 748-AAF | Krios 3 | 0.825 | 664 | 0.52 | 47.4 | Y | Y | 416 |
| 748-ADP | Krios 2 | 0.849 | 664 | 1.46 | 175.9 | Y | Y | 416 |
| 755-ANP | Krios 1 | 1.072 | 512 | n.c. | n.c. | Y | Y | 320 |
| 755-AAF | Krios 2 | 0.848 | 664 | 1.6 | 179.2 | Y* | Y* | 416 |
| 755-ADP | Krios 1 | 0.831 | 664 | 0.35 | 70 | N | N | 416 |
| 755 Apo | Krios 2 | 1.088 | 512 | 1.5 | 171.7 | N | N | 320 |
| 772-ANP | Krios 1 | 1.073 | 512 | n.c. | n.c. | N | N | 320 |
| 772-AAF | Krios 3 | 0.826 | 664 | 0.44 | 49.6 | Y | Y | 414 |
| 772-ADP | Krios 2 | 0.849 | 664 | 1.46 | 3.6 | N | N | 416 |
| 772 Apo | Krios 2 | 0.850 | 664 | 1.25 | 3.1 | N | N | 416 |

Notes

^a^ Given when estimated and corrected for a given dataset.

n.c.: magnification anisotropy not corrected.

* Dataset for which this step was performed after the final helical reconstruction and before the helical assembly subunit refinement and classification (HASRC) procedure.
