# Supplementary material for: Structural basis of mechano-chemical coupling by the mitotic kinesin KIF14
Source: Nat Commun. 2021 Jun 15;12:3637. doi: 10.1038/s41467-021-23581-3 (PMC8206134; doi:10.1038/s41467-021-23581-3)
Supplement: Supplementary file 7 — Source Data [file 41467_2021_23581_MOESM7_ESM.zip › DataSource/Table_2_KIF14_NBP_distances_etc.doc]

|  | **Nucleotide** | **MT-bound** | **Selected NBP distances (Å)** | | **Cβ-twist** | **Neck-linker** | **Model** | **PDB ID** |
| --- | --- | --- | --- | --- | --- | --- | --- | --- |
|  |  |  | K488-R604 (SW1-PL) | R406-T647 (KH0-SW2) | KS3 ∠ KS4 |  |  |  |
| **Semi-Closed** | ADP | No | **13.4** | **21.4** | **45.8** | Undocked, mobile | K-ADP | 4OZQ |
|  |  |  |  |  |  |  |  |  |
| **Open** | ADP | Yes | 14.3 | 24.9 | 44.6 | Undocked, mobile | MT-K743-ADP | 6WWQ |
|  | ADP | Yes | 14.8 | 23.7 | 42.8 | Undocked, mobile | MT-K748-ADP | 6WWM |
|  | ADP | Yes | 14.4 | 24.5 | 46.0 | Undocked, sideways | MT-K755-ADP | 6WWJ |
|  | ADP | Yes | 14.8 | 22.4 | 42.2 | Undocked, sideways | MT-K772-ADP | 6WWF |
|  | Apo | Yes | 14.7 | 23.0 | 45.4 | Not present | MT-K735-Apo | 6WWT |
|  | Apo | Yes | 14.1 | 23.6 | 42.7 | Undocked, mobile | MT-K743-Apo | 6WWP |
|  | Apo | Yes | 14.6 | 24.9 | 44.4 | Undocked, sideways | MT-K755-Apo | 6WWI |
|  | Apo | Yes | 15.2 | 25.4 | 41.8 | Undocked, sideways | MT-K772-Apo | 6WWE |
|  |  | **Avg** | **14.6** | **24.0** | **43.7** |  |  |  |
|  |  | **sd** | 0.3 | 1.1 | 1.6 |  |  |  |
|  |  |  |  |  |  |  |  |  |
| **Open^*^** | AMP-PNP | Yes | 14.2 | 20.9 | 44.0 | Not present | MT-K735-ANP | 6WWV |
|  | AMP-PNP | Yes | 14.3 | 24.2 | 45.6 | Undocked, mobile | MT-K743-ANP-O | 6WWS |
|  | AMP-PNP | Yes | 14.3 | 24.4 | 44.1 | Undocked, backwards | MT-K755-ANP-L | 6WWL |
|  | AMP-PNP | Yes | 14.0 | 22.2 | 43.3 | Undocked, backwards | MT-K772-ANP-L | 6WWH |
|  | ADP-AlFₓ | Yes | 14.4 | 23.5 | 42.6 | Not present | MT-K735-AAF | 6WWU |
|  | ADP-AlFₓ | Yes | 14.3 | 23.8 | 45.1 | Undocked, mobile | MT-K743-AAF-O | 6WWR |
|  | ADP-AlFₓ | Yes | 14.8 | 22.8 | 44.0 | Undocked, backwards | MT-K755-AAF-L | 6WWK |
|  | ADP-AlFₓ | Yes | 14.5 | 21.8 | 44.7 | Undocked, backwards | MT-K772-AAF-L | 6WWG |
|  |  | **Avg** | **14.3** | **22.9** | **44.2** |  |  |  |
|  |  | **sd** | 0.2 | 1.2 | 1.0 |  |  |  |
|  |  |  |  |  |  |  |  |  |
| **Closed** | AMP-PNP | Yes | 12.7 | 12.0 | 36.4 | Docked ^†^ | MT-K743-ANP-C | 7LVQ |
|  | AMP-PNP | Yes | 12.8 | 11.2 | 37.2 | Docked | MT-K748-ANP | 6WWO |
|  | AMP-PNP | Yes | 12.3 | 11.0 | 36.4 | Docked, partially unzipped | MT-K755-ANP-T | 6WWL |
|  | AMP-PNP | Yes | 12.3 | 13.0 | 35.0 | Docked, partially unzipped | MT-K772-ANP-T | 6WWH |
|  | ADP-AlFₓ | Yes | 13.1 | 11.2 | 33.7 | Docked  ^†^ | MT-K743-AAF-C | 7LVR |
|  | ADP-AlFₓ | Yes | 12.4 | 11.6 | 35.4 | Docked | MT-K748-AAF | 6WWN |
|  | ADP-AlFₓ | Yes | 11.9 | 11.9 | 37.0 | Docked, partially unzipped | MT-K755-AAF-T | 6WWK |
|  | ADP-AlFₓ | Yes | 12.8 | 11.4 | 36.7 | Docked, partially unzipped | MT-K772-AAF-T | 6WWG |
|  |  | **Avg** | **12.5** | **11.7** | **36.0** |  |  |  |
|  |  | **sd** | 0.4 | 0.6 | 1.2 |  |  |  |
